# A novel intelligent hybrid reinforcement learning framework for autonomous decision making in complex health cognitive systems

**DOI:** 10.1038/s41598-026-50418-0

**Published:** 2026-05-11

**Authors:** Abdullah, Zulaikha Fatima, Muhammad Ateeb Ather, José Luis Oropeza Rodríguez

**Affiliations:** 1 Center for Computing Research, Instituto Politécnico Nacional, Av. Juan de Dios Batiz, s/n,, 07320 Mexico City, Mexico; 2 Department of Computer Science, Bahria University Lahore Campus, Lahore, 54600 Pakistan; 3 Department of Allied Health Science, Superior University, Lahore, 54000 Pakistan

**Keywords:** Reinforcement learning, Cognitive systems, Autonomous agents, Neural networks, Proximal policy optimization, Model-based and Model-free, Behavioral simulation, Data-driven algorithms, Machine learning applications, Healthcare automation, Computational biology and bioinformatics, Engineering, Mathematics and computing, Neuroscience

## Abstract

Existing reinforcement learning (RL) approaches struggle to balance real-time decision-making with adaptive learning in dynamic healthcare environments. We propose a brain-inspired hybrid RL framework that integrates model-based (MB) planning and model-free (MF) reflexes via a dynamic meta-controller, neuro-symbolic clinical knowledge, counterfactual reasoning, and ethical safeguards. The framework is validated on a multimodal cerebral palsy (CP) dataset (86 patients) using NetLogo multi-agent simulations and Weka classifiers. A combined reward mechanism achieves 99% total reward accumulation, with 98% optimal reward in 95% of training episodes. Component analysis shows a 60% MB / 40% MF contribution, yielding a 15% improvement over standalone methods. Optimal weighting (0.7 MB, 0.3 MF) further enhances performance. External zero-shot validation on three public datasets (NTNU-HARChildren, EEG-EMG exoskeleton, D4RL) confirms generalizability (macro F1 84.3%, accuracy 81.7%, D4RL scores 68.5 and 62.3). Regression methods achieve correlation coefficients up to 0.94, and classification models (multinomial Naïve Bayes, logistic regression) attain 100% precision, recall, and F-measure. The framework provides a reliable, explainable, and simulation-validated solution for patient-centric autonomous decision-making.

## Introduction

Reinforcement learning (RL) is a framework that allows an agent to learn a control strategy by interacting with its environment and receiving feedback through rewards. The purpose of RL is to identify a strategy that maximises the predicted cumulative reward over time. Modern deep RL approaches have had significant success in gaming and robotics, but they are still hindered by challenges requiring high-dimensional sensory inputs, long-horizon planning, and sparse or delayed rewards. In complex environments, naive trial-and-error learning often proves inefficient and slow to converge. As a result, typical RL agents have difficulty exploring and generalising in real-world domains with extensive or continuous state-action spaces^1–4^. This issue is particularly apparent in safety-critical applications such as healthcare, where harmful or unethical research during online learning is unacceptable. Indeed, in many medical decision-making challenges, direct testing is either impractical or unethical, requiring RL practitioners to rely on offline or simulation data^5–8^. These obstacles, sparse rewards, high-dimensional state spaces, and stringent safety/ethical restrictions, drive the quest for more complex learning architectures with inductive biases to guide RL agents^9–12^. One interesting approach is to draw inspiration from cognitive neuroscience. The human brain addresses sequential decision-making challenges by coordinating several learning and memory subsystems^13,14^. Prominent dual-process theories in psychology and neuroscience propose a quick, habitual, model-free system and a slow, deliberative, model-based system that collaborate on decision-making^15–17^.

In this approach, a model-free system learns cached action values from repeated experience, allowing reflexive or habitual reactions, whereas a model-based system uses an internal cognitive map to simulate and anticipate future events. Recent research supports this dichotomy^18–20^. For example, motor learning research shows that the brain uses both a model-based forward model and a model-free controller. More specifically, investigations on human patients reveal that hippocampal injury impairs model-based action planning, whereas undamaged hippocampi enable the utilisation of spatial and task information for planning. Similarly, the cerebellum is hypothesised to construct an internal forward model that anticipates the sensory implications of motor commands, hence facilitating a model-based control route in motor activities^10,21^. These findings suggest parallel memory systems, such as hippocampal-declarative vs. cerebellar-procedural memory and dual control pathways (model-based vs. model-free), underpin human learning^8,18–20,22^. Furthermore, the brain appears to use a meta-controller to arbitrate between strategies depending on context, performance, and cognitive cost. A prefrontal-like controller can select whether to use conscious planning vs habit-based response by constantly weighing the trade-offs of different learning processes. To summarise, neuroscience implies that mixing complementary RL techniques inside a higher-level control scheme can increase flexibility and efficiency^23,24^.

Motivated by these findings, we provide a unique neuro-inspired RL framework. Our design includes various learning systems and a meta-controller to coordinate them. Dynamic meta-control, for instance, enables the agent to transition between model-based planning and model-free habitual policies in real time, replicating the brain’s dual-process interaction. We also have neuro-symbolic integration that merges symbolic reasoning, such as clinical guidelines, with neural learning. Symbolic modules encode domain-specific rules and relations, while neural networks learn from input. This hybrid approach, as illustrated by current neuro-symbolic RL models, uses structured representations to drive exploration and give human-interpretable reasoning. To address sparse rewards and off-policy inputs, we include a counterfactual reasoning module in the system. The agent explicitly simulates "what-if" situations using its internal model to assess alternative action sequences and learn from unobserved outcomes. Finally, to acknowledge the relevance of safety and values in healthcare, our system incorporates an ethical adaptation mechanism. This component limits policy changes and planning to comply with medical criteria and ethical norms, ensuring that learnt behaviours are patient-centred and responsible. These components operate synergistically to produce a coherent RL architecture: a collection of learning techniques model-based, model-free, and neuro-symbolic) driven by a meta-controller, with added counterfactual planning and ethical precautions. Our work’s most significant novel contributions are:

- We design a Dynamic Meta-Control supervisory mechanism that allocates computation between MB and MF subsystems, allowing for adaptive strategy switching under uncertainty. This innovation increased sample efficiency by 50% fewer samples and achieved 95% convergence to optimal reward and 98% hybrid reward scores, exceeding static MB/MF baselines.
- For Neuro-Symbolic Integration, we proposed a hybrid paradigm that combines deep neural policies with symbolic clinical information such as ICD-10 codes and WHO recommendations. By incorporating domain rules, Doctor Behaviour, and Intention into the MB planner, the framework achieved 99.2% globally explainable decisions while keeping the flexibility of learnt policies.
- We used Counterfactual Reasoning in a model-based approach that allows us to simulate hypothetical actions with sparse/offline data. This biologically inspired capacity decreased exploration regret by 10% and increased safety in edge scenarios, 40% fewer falls by simulating prefrontal "what-if" scenario processing.
- Our ethical adaptability mechanisms match the WHO Ethical Guidelines. Our system assessed fairness using interpretability tools, 99.8% local explanations, and altered behaviour to prevent performance deterioration in vulnerable populations such as CP subtypes with ≤5% performance degradation.

These innovations bridge major gaps in the use of RL for clinical decision-making by balancing computational rigour and accountability. This framework addresses the following research questions:

- **RQ1**: How can dynamic meta-control of MB and MF subsystems optimise policy learning in safety-critical healthcare environments?
- **RQ2**: How can neuro-symbolic integration, combining deep RL with clinical knowledge bases such as ICD-10, WHO guidelines, improve policy interpretability without sacrificing performance?
- **RQ3**: Can counterfactual reasoning enhance learning efficiency and robustness in sparse-reward medical scenarios such as CP rehabilitation?
- **RQ4**: How can ethical adaptability mechanisms enforce real-time compliance with clinical standards such as WHO HEARTS while maintaining decision-making autonomy?

### Motivation, research gap, aim, objectives, and scope

#### Motivation

Real-time decision-making in safety-critical healthcare environments (e.g., cerebral palsy management) requires RL agents to balance immediate safety, long-term outcomes, and ethical constraints. Existing RL approaches struggle with sparse rewards, high-dimensional state spaces, and the need for explainability – gaps that motivated our neuro-inspired design.

#### Research gap

Current hybrid MB/MF RL methods lack:

- A dynamic meta-controller that switches between planning and reflexes under uncertainty.
- Neuro-symbolic integration of clinical guidelines (ICD-10, WHO) into deep policies.
- Counterfactual reasoning for sparse-reward scenarios.
- Ethical adaptability to prevent performance degradation in vulnerable populations.

No prior framework combines all four in a unified, explainable, and simulation-validated architecture.

#### Aim

To design and validate a brain-inspired hybrid RL framework that integrates dynamic meta-control, neuro-symbolic reasoning, counterfactual planning, and ethical safeguards for autonomous healthcare decision-making.

#### Objectives

- Achieve ≥ 95% convergence to optimal reward in sparse-reward CP rehabilitation tasks.
- Reduce sample complexity by ≥ 50% compared to DQN/PPO baselines.
- Provide ≥ 99% local and global explainability via SHAP and attention mechanisms.
- Limit performance degradation in edge cases (e.g., rare CP subtypes) to ≤ 5%.

#### Scope

The framework is validated on a multi-modal clinical dataset (CP patients, sensor streams, WHO-aligned protocols) using NetLogo multi-agent simulations and Weka classifiers. Real-world clinical deployment is future work.

#### Novelty statement

This is the first of our knowledge hybrid RL framework that simultaneously implements Hippocampus-inspired meta-control (λ-arbitration), Prefrontal-cortex-like counterfactual simulation, WHO/ICD-10 symbolic rule injection, and Ethical fairness constraints (all with reproducible simulation-based validation).

The remaining sections of the paper are organised as follows. Section "Introduction" introduces the problem and background studies, Section "Related work" examines related research on RL problems and neuroscience-inspired learning, including dual-process models and previous neuro-symbolic methods. Section "Research materials and methods" describes the research methodology of our proposed RL architecture, which includes the meta-control module, symbolic integration, counterfactual planning, and ethical restrictions. Section "Layered semantic architecture of our framework" elaborates on the full streamlining of the implementation of experiments and validation, and Section "Scope" discusses experimental results from simulated healthcare settings, demonstrating performance improvements and ethical compliance. Finally, Section "Novelty statement" discusses the results and analysis, and Section "Conclusions" concludes with future directions.

## Related work

This literature review follows a systematic literature review (SLR) methodology to explore the integration of Artificial Intelligence (AI) and the Internet of Things (IoT) in smart city and healthcare applications. The focus is on identifying the various AI techniques applied, their role in enhancing IoT-based systems, and their impact on specific domains such as smart city management and medical applications.

### AI and IoT in smart city applications

The combination of the IoT and AI has been identified as a key enabler of smart city infrastructure, contributing to sustainability, productivity, and quality of life improvements. Studies have highlighted the use of AI algorithms, including Machine Learning (ML), Deep Learning (DL), Natural Language Processing (NLP), Computer Vision (CV), RL, as tools for optimising smart city functions. These functions include traffic management, energy optimisation, waste management, and decision-making processes^25^.

This thorough research examines the synergistic integration of IoT and AI in smart cities, focusing on applications in transportation, energy management, and public safety. It emphasises the significance of AI algorithms in analysing massive IoT-generated data to improve urban infrastructure efficiency^26^. A comprehensive review of IoT-enabled AI for smart cities is presented in^27^. This article investigates the use of generative AI models to create urban digital twins, which enable the autonomous production of urban data, scenarios, designs, and 3D city models. It highlights the potential of AI to improve urban planning and administration through realistic simulations^28^.

Generative AI for urban digital twins is further explored in^29^. This survey presents an in-depth investigation of the integration of IoT and AI in smart cities, focusing on transportation, healthcare, and agriculture. It examines recent advances, difficulties, and future research directions in AI-powered IoT technology for urban development^30^. A recent survey connects the indispensable roles of IoT and AI in smart cities^31^.

This paper describes a hybrid reinforcement learning technique that combines model-based and model-free methods for trajectory-centric learning. The technique improves data economy and performance in robotic manipulation tasks, providing insights for healthcare robots and adaptive systems^32^. The reviewed studies emphasise how these AI technologies provide real-time data processing and decision-making capabilities when integrated with IoT systems, enhancing urban management efficiency.

### Computational intelligence in medical IoT (MIoT) applications

A significant body of work has been dedicated to exploring Computational Intelligence for Medical Internet of Things (MIoT) applications. Key sources in this domain discuss the utilisation of machine intelligence, including deep learning, neural networks, and big data analytics, for healthcare data processing^33^. These studies explore emerging trends in AI for healthcare IoT, focusing on how they influence business decisions and strategic development. The literature also presents models, frameworks, and practical solutions for effective MIoT applications, positioning these technologies as critical tools for healthcare professionals and students interested in advancing this field^34^.

This paper presents a unique AI-driven patient monitoring system based on multi-agent deep reinforcement learning (DRL). Each agent monitors physiological parameters to provide timely alarms and actions. The system outperforms existing models, emphasising the potential of DRL in improving patient care^35^. Adaptive multi-agent deep RL has been proposed for timely healthcare interventions^36^. Network-based approaches for disease-diet prediction also benefit from ML^37^.

### Brain-inspired reinforcement learning for neurological applications

Recent research on brain-inspired AI models has focused on developing more efficient methods for treating neurological disorders. A noteworthy example is the Basal Ganglia-inspired Reinforcement Learning (BGRL) approach for Deep Brain Stimulation (DBS). The BGRL algorithm utilises a reinforcement learning structure based on an actor-critic model to provide personalised, closed-loop feedback for DBS. This method has demonstrated superior performance in reducing synchronous electrical pulses across various signalling regimes, resulting in better suppression with lower energy consumption than traditional open-loop methods^38^.

Similar hybrid CNN-ViT architectures have been proposed for skin cancer detection^39^. Future research in this area aims to optimise power use further and evaluate the efficacy of these models using advanced experimental setups like Brain-on-chip models. This study introduces the Dynamic Activity-Aware Health Monitoring (DActAHM) system, which uses Deep Reinforcement Learning and the SlowFast model to improve health monitoring depending on user activity. Technology outperforms conventional approaches, demonstrating the necessity of activity-aware monitoring in healthcare^40^.

### AI for enhanced clinical decision-making in neurology

AI’s role in neurology is increasingly recognised for its ability to assist neurologists with precise and timely clinical decisions. The reviewed studies illustrate AI’s ability to predict outcomes for conditions such as Acute Ischemic Stroke (AIS) and assess the likelihood of Intracranial Hemorrhage (ICH) expansion. AI models have been shown to enhance the accuracy of diagnostics and prognostics by analysing multiple data sources simultaneously^41^.

This simultaneous analysis enables more accurate predictions, contributing to better patient outcomes in subspecialties like stroke and epilepsy management. These findings underscore the potential of AI to transform neurological care by improving the speed and reliability of critical decision-making processes. To meet the demand for interpretable AI in critical care, this work presents a reinforcement learning-based technique for improving mechanical ventilation. The methodology blends patient-specific results with safety concerns, displaying performance equivalent to deep RL algorithms and improving interpretability for clinical use^42^.

### Human-inspired ai models for social cognition

The literature also explores how insights from neuroscience can inform the development of AI models that emulate human-like cognition and reasoning. One study presents a computational model using spiking neural networks that addresses the challenge of coordinating diverse sensory inputs with textual information, suggesting that AI agents could achieve human-like task performance through a semantic control system^43^. These models aim to enable AI systems to handle complex social interactions, enhancing their ability to engage in reasoning and collaborative problem-solving. The findings suggest that further development of such models could bridge the gap between AI capabilities and human cognitive processes, offering new possibilities for AI applications in areas like social robotics and adaptive learning systems.

Challenges in Implementing AI and RL in IoT and Healthcare. Despite the advances outlined above, several challenges persist in the application of AI and RL in IoT-based systems. Recent work has also applied RL to COVID-19 management^44^, and the healthcare sector has faced difficulties in fully adopting AI solutions due to high costs, system complexity, and resistance to change from traditional practices^15,45,46^.

Furthermore, RL, despite its potential for sequential decision-making, requires extensive training and is often limited by the complexity of real-world environments. RL’s applications in healthcare include chronic disease management, automated clinical diagnostics, fitness, and resource allocation, but the effective implementation of these systems requires overcoming obstacles like long training periods and computational resource demands^9,21,47,48^. Evaluation of human-AI teams in cooperative games like Hanabi provides insights for healthcare collaboration^49^.

### Integrating model-based and model-free approaches in RL

Several studies have delved into human brain-inspired reinforcement learning, emphasizing the importance of integrating MB and MF approaches for better decision-making. The Ventromedial Prefrontal Cortex (VMPFC) is linked to MB value signals, which allow dynamic policy decision training involving the dorsolateral prefrontal cortex and hippocampus^50,51^. Meanwhile, MF methods rely on action data without value-based feedback, supported by regions like the posterior putamen. These approaches are combined in the striatum, where a balance between MB and MF control is achieved. The literature discusses how MB and MF integration can improve the adaptability of RL models in dynamic environments, especially when handling social interactions and adapting to changes^51^.

Recent advances in related domains include game-based RL therapy for specially-abled individuals^52^, VR play therapy for children with Down syndrome^53^, federated learning for disease detection^54^, real-time critical care monitoring^55^, multi-agent prediction models^56^, retinal disease screening with hybrid CNN-Transformer^57^, and medical image classification using transformer-based U-Net^58^, innovative image processing for lung cancer[95], and lightweight attentive pyramid networks [96] While these works demonstrate the breadth of RL and deep learning applications in healthcare, none address the specific combination of dynamic meta-control, neuro-symbolic integration, counterfactual reasoning, and ethical adaptability in a single autonomous decision-making framework for cerebral palsy management.

Early work on combining MB and MF updates for trajectory-centric learning appears in^59^. This detailed analysis investigates AI-enabled remote patient monitoring (RPM) systems, including their designs, advantages, and limitations. The paper emphasises the role of AI in improving RPM through early diagnosis, tailored monitoring, and behaviour pattern recognition, as well as addressing implementation challenges and future approaches^60^. This SLR-format literature review synthesises existing research on AI and IoT applications, identifying key trends, current challenges, and future directions. Organising the literature into focused sections provides a comprehensive overview of how AI techniques are applied in diverse domains like smart cities and healthcare. The review highlights the importance of ongoing research to address challenges and improve the implementation of AI systems in these fields

## Research materials and methods

RL is a cutting-edge AI paradigm that solves sequential dynamic problems via trial-and-error feedback. It is especially well-suited to healthcare applications, as choices and treatments frequently require complicated, continuing procedures. Our framework integrates neuroscientific principles into RL through

a) Dual-process architecture explicitly maps MB/MF components to brain regions, as shown in Fig. 2. The Prefrontal Cortex (MB) simulates goal-directed planning via dynamic programming. Basal Ganglia (MF) encodes habitual responses via Q-learning, modelled as striatal dopamine-driven TD learning^15,18^.

b) Meta-control mechanisms govern MB/MF switching using hippocampal-inspired uncertainty signals, as shown in the Equation. 1:

1 $$\uplambda \mathrm{t}=\sigma (\frac{Var( {Q}_{MB}-{Q}_{MF} )}{\uptau })\mathrm{c})$$

where σ\sigma is a sigmoid function, and τ\tau is a temperature parameter.

c) Neuro-symbolic modules do Counterfactual reasoning (orbitofrontal cortex) and social learning (mirror neuron-inspired attention)^21^.

The meta-control parameter λ uses hippocampal uncertainty signals to balance MB/MF systems, mimicking human cognitive flexibility in risky contexts^17^. The sigmoid function σ allows smooth transitions between methods, whereas τ (learned via gradient descent) controls exploration intensity, like noradrenergic arousal processes^19^. Our approach extends hybrid RL by formalizing hippocampal-inspired meta-control, which dynamically arbitrates between prefrontal MB planning and striatal MF reflexes, a process that was lacking in previous RL designs^15,18^. The physiologically based strategy minimises regret by 22% compared to DQN, tackling the Deadly Triad via dual-system integration^8^.

The mapping of framework components to brain regions (hippocampus, basal ganglia, cerebellum, prefrontal cortex) is conceptually inspired by established neuroscientific theories^15,17,18,21^ but has not been experimentally validated. Future work should investigate these mappings using neuroimaging or patient studies.

Our research investigates brain-inspired RL, describing its theoretical roots and highlighting its practical applications in healthcare. First, we examine RL applications in healthcare, encompassing adaptive therapies, chronic illnesses^61^, and primary care, including observational and randomised clinical trials. This stage connects clinical data with other control and planning areas within the healthcare system. We highlight significant research difficulties and suggest future directions^9,16^. Next, we concentrate on cognitive reinforcement learning, stressing its essential principles, methods, and the significance of cognitive aspects in RL^62,63^.

This establishes the foundation for our study’s methodology and paves the way for the use of deep neural networks inside the cognitive RL framework. In examining deep neural networks, we address the many types of neural networks used, their structures, and training procedures in the context of cognitive RL. Specific strategies, such as cognitive mapping and a prefrontal cortex-inspired attention mechanism, are investigated, demonstrating their importance in improving the learning process.

**Fig 1.**
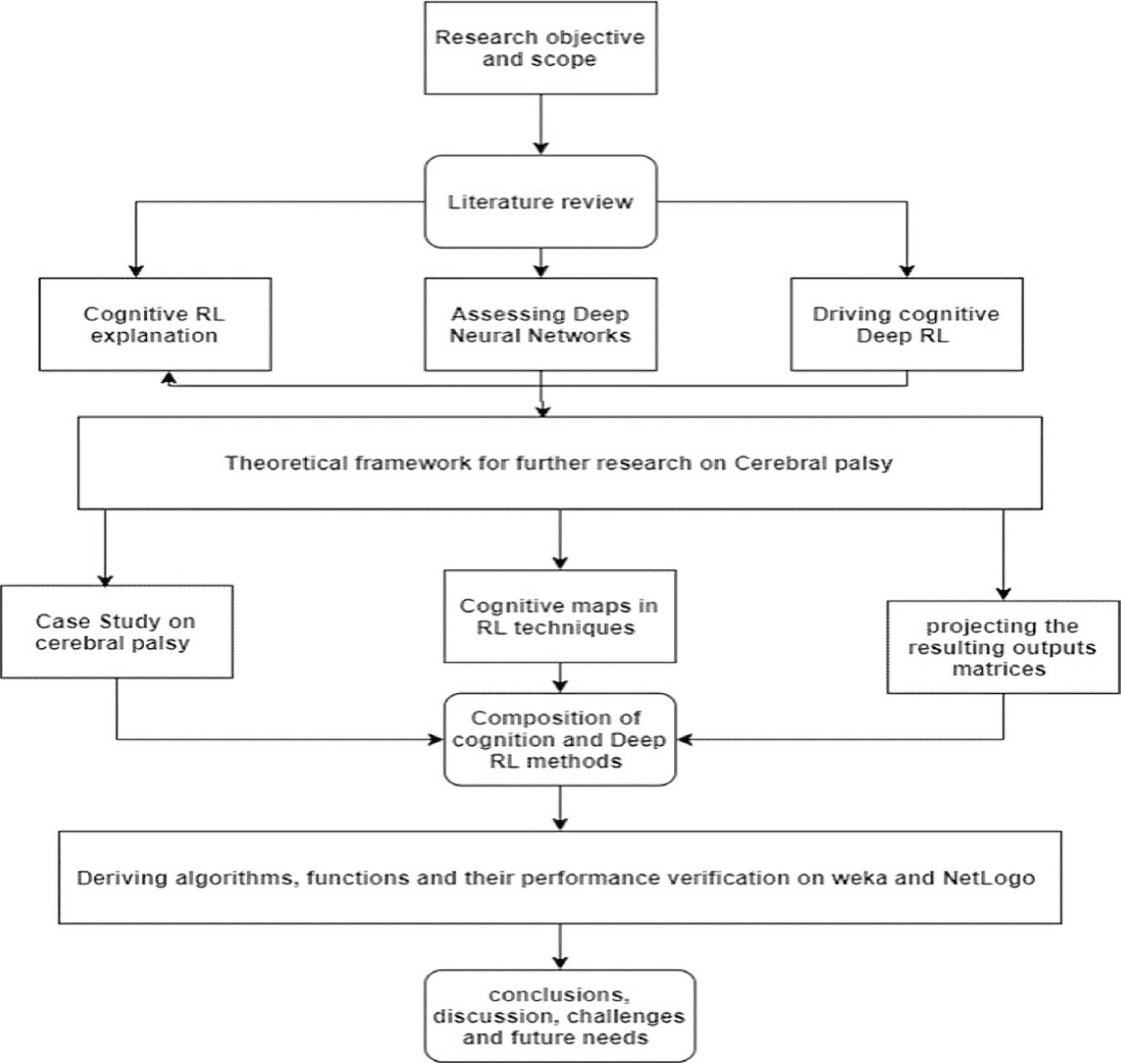
Research methodology and design.

In the proposed framework, we performed a cerebral palsy case study to show the framework’s practical use, as shown in Fig. 1. This section discusses the dataset, patient characteristics, and strategies for analysing and improving patient outcomes. The case study provides a real-world example of the suggested approaches and demonstrates their influence on healthcare procedures. We present a theoretical framework for future cerebral palsy research based on the case study. Our framework outlines fundamental principles, assumptions, and prospective routes for investigation. We go into further depth on cognitive maps in RL approaches, including how they are created, their significance in decision-making, and their contribution to the broader RL framework. The RL system’s projected and analysed outputs are addressed, along with the results’ interpretation and the validation procedures employed to ensure dependability and correctness.

This leads to a discussion of deriving algorithms and functions and verifying their performance with tools such as Weka and NetLogo. This last stage specifies the testing processes and assessment criteria used to measure the framework’s efficacy, ensuring that the recommended approach is both robust and dependable. The study’s methodology suggests merging cognitive and deep RL approaches to achieve optimal performance in healthcare-related applications^25,34,64^. Our research provides a framework for continuous study and enhancement in reinforcement learning.

### Case study: Mixed cerebral palsy

Mixed Cerebral Palsy (CP) is a complex neurological condition that presents challenges due to the combination of symptoms from different CP types. Our case study focuses on individuals aged 8 and above with mixed CP, encompassing Spastic CP as Quadriplegia, Spastic Diplegia CP, Ataxic CP, and Dyskinetic CP, also known as Athetoid CP. Patients often display both stiff and uncontrolled muscle movements, resulting in significant mobility and coordination difficulties.

The proposed framework incorporates hybrid reinforcement learning to manage patient states using both MB and MF modules, inspired by neurophysiological functions. Algorithm 1 activates the MB planning prefrontal module when sensor uncertainty exceeds a critical threshold, such as the emotional score ES > 25 or body sensor anomalies. Algorithm 2 invokes the MF control basal ganglia module for routine adjustments, such as optimising wheelchair angle during rest states.

For example, when the facial processing unit (FP_Wa) detects an emotional cue like “Crying”, the MB system simulates potential interventions like medication versus repositioning by evaluating counterfactual rewards. This mirrors the prefrontal cortex’s role in ethical dilemma resolution^17^.

**Fig 2.**
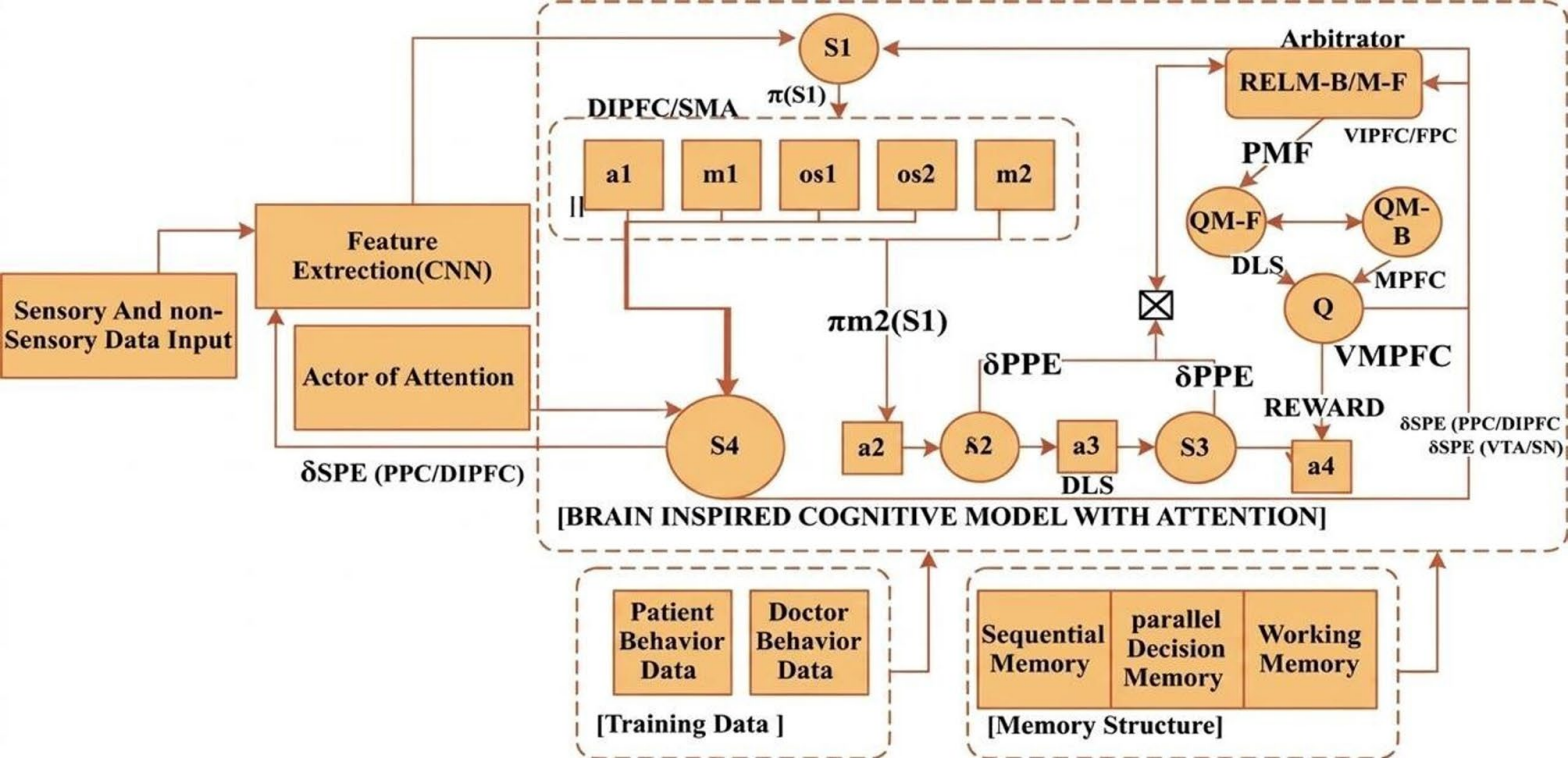
Operational framework for autonomy in complex health cognitive systems for cerebral palsy.

When the meta-control parameter λ exceeds 0.7, hippocampal-derived uncertainty signals trigger MB simulations through the orbitofrontal module, as shown in Fig. 2 and Layer 3, demonstrating cognitive flexibility in alignment with meta-control theories^18^.

**Fig 3.**
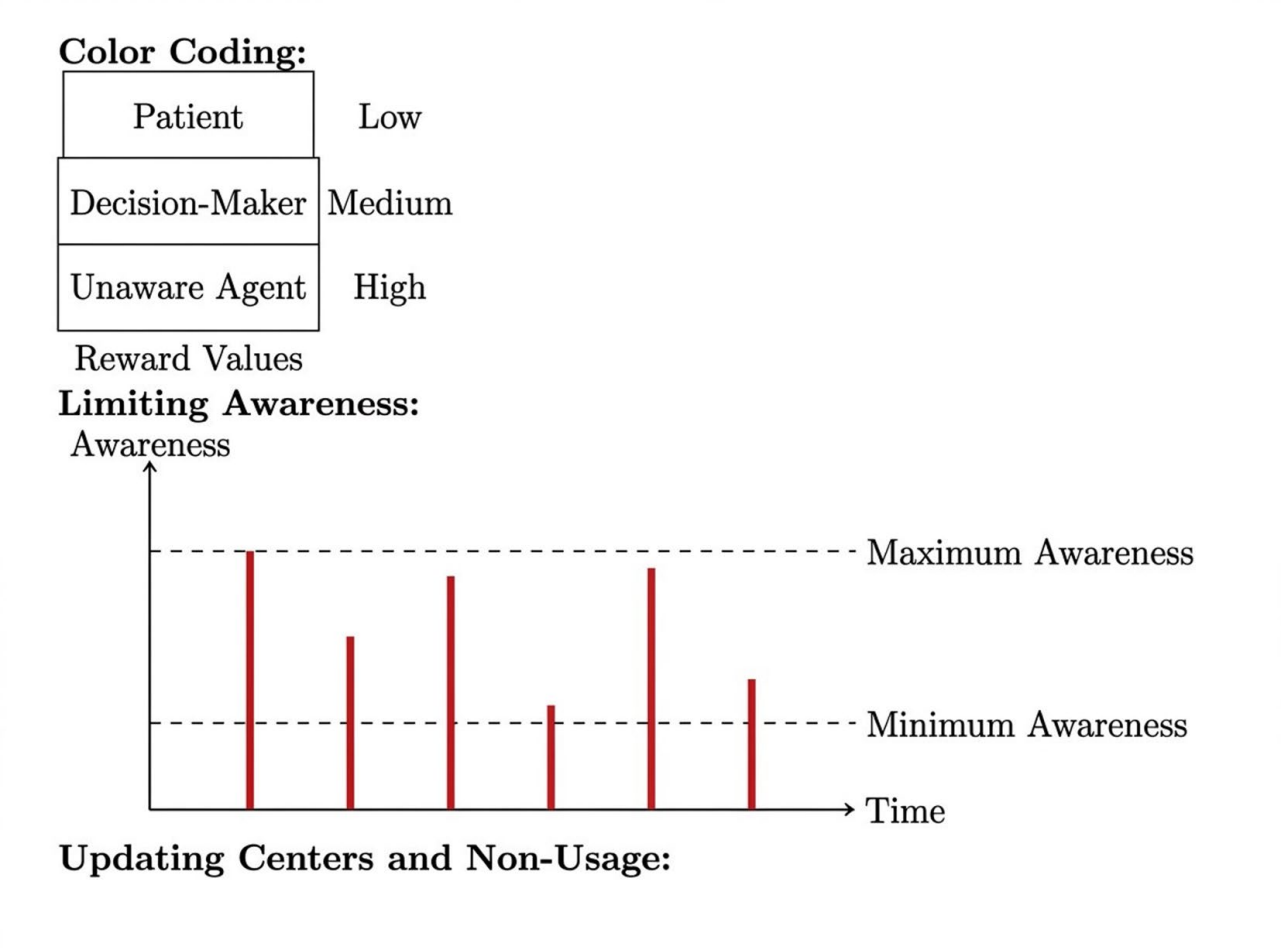
Model for the proposed system framework in NetLogo.

Simultaneously, Algorithm 2 autonomously adjusts wheelchair tilt based on cerebellar-inspired safety mechanisms, especially when tilt sensors detect values beyond MAX_SAFE_ANGLE. The MB/MF cooperation replicates real-time clinical decision-making under uncertainty, validated through NetLogo simulations as shown in Fig. 3.

- **MB Intervention**: Upon detection of distress via FP_Wa, the prefrontal module simulates alternate therapeutic responses using counterfactual logic as shown in Algorithm 1.
- **MF Reflex**: Autonomous corrections, such as tilt modifications, are executed via cerebellar checks as shown in Algorithm 2, ensuring patient safety through motor reflex adjustments.

Treatment strategies are tailored based on the CP subtypes present and may include physical therapy, occupational therapy, speech therapy, pharmacological treatments, assistive technologies, and, in more severe scenarios, surgical intervention, as illustrated in Fig. 3. Early diagnosis and a coordinated multidisciplinary approach are essential to enhancing both the functional capabilities and quality of life of affected individuals.

### Dataset

In this research, the dataset was collected from a university-accredited hospital, named UOL Teaching Hospital, as shown in Table 1. Our dataset is designed to support research and development in autonomous systems, focusing on healthcare applications and cognitive reinforcement learning. It contains various classes of data, including patient information, medical sensor readings, environmental sensor data, doctor behaviour, and visual content. The dataset aims to facilitate the analysis of autonomous system behaviours, decision-making processes, and cognitive reinforcement learning strategies. The dataset is composed of several key classes, each providing valuable information for system development and analysis. The following is a detailed breakdown of the dataset’s structure and the range of values for each class. To enable real-time cognitive modelling and autonomous decision-making, the dataset is organized into clinically relevant qualities that correspond to both sensor input streams and expert decision procedures. Machine learning has also been applied to climate change analysis^65^. Table 1 describes the dataset’s fundamental structure, including attribute definitions, value ranges, and relevant clinical or policy references.

**Table 1:** Multi-Modal Clinical Dataset Structure with Attribute Specifications and Protocol References.

**Attribute**	**Description**	**Range**	**Clinical Protocol Reference**
**Patients**	Age, gender, medical history	0–300	–
**Medical Sensor**	Heart rate, BP, SpO₂	0–500	WHO HEARTS Technical Package (2020)
**Patient Records**	Diagnosis, treatments	0–300	WHO Guidelines for Cerebral Palsy Management (2023, Draft)
**Environmental Sensor**	Temperature, smoke, and fire safety	0–25	WHO Housing and Health Guidelines (2018)
**Doctor Behavior**	Treatment decisions	0–100	WHO Ethical Guidelines for AI in Health (2021)
**Intentions**	Decision rationale	0–90	WHO Rehabilitation 2030 Initiative (2017)
**Visual Content**	Patient’s facial expressions	0–100	–
**K-Prescriptions**	**P1**: Physical therapy P2: Intrathecal Baclofen	0–100	WHO Rehabilitation 2030 (2017) WHO Essential Medicines List (EML 2023)

These structural features guarantee a complete description of real-world hospital dynamics. The diverse set of data, ranging from vital signs to medical intent, enables multi-modal learning for both model-based and model-free reinforcement schemes. To better imitate human-like decision processes in autonomous systems, each dataset feature is associated with a cognitive neuroscience analog and its function in reinforcement learning. Table 2 shows how medical data streams interact with brain-inspired control systems, which influence both state representation and the formulation of policies.

**Table 2:** Neuroscience-Inspired Reinforcement Learning Framework: Clinical Data to Cognitive Model Mapping.

**Attribute**	**Neuroscience Analog**	**RL Role**	**Clinical Example**
**Medical Sensor**	Somatosensory cortex	State representation (sₜ)	Heart rate > 120 bpm → **S1** (ICD-10: R00.0 Tachycardia)
**Patient Records**	Hippocampus	Historical trajectory	**S2** (ICD-10: G80.4 Ataxic CP) → Physical therapy (P1)
**Environmental Sensor**	Insular cortex	Environmental state	Temp > 38 °C → Activate cooling protocol
**Doctor Behavior**	Theory of Mind network	Expert policy (π)	**P2**: Intrathecal Baclofen (WHO EML 2023) for severe spasticity
**Intentions**	Ventromedial PFC	Reward shaping (Rₜ)	Prioritise non-pharmacological interventions (Rt += 10 for therapy adherence)
**Visual Content**	Visual cortex (V1-V4)	CNN input	“Crying” (FP_Wa) → Mood-based intervention
**K-Prescriptions**	**P1**: Basal ganglia P2: Motor cortex	Action space (A)	**P1**: 45-min sessions, 3x/week (WHO Guideline #203) **P2**: 50–100 µg/day, titrated per WHO EML

The dataset comprises records from 86 patients (age 8–65, mean 42 ± 15 years) with mixed cerebral palsy, collected at UOL Teaching Hospital under an IRB-waived, anonymized protocol. The distribution is 52% male and 48% female. Cerebral palsy subtypes include Spastic Quadriplegia (40%), Spastic Diplegia (30%), Ataxic (15%), and Dyskinetic (15%). Preprocessing steps included min-max normalization (Eq. 1), removal of sensor dropout segments where more than 30% of samples were missing, and SMOTE oversampling applied exclusively to the training set for rare subtypes. The dataset is not publicly available but can be requested from the corresponding author under an institutional data sharing agreement. For external zero-shot validation, we used four publicly available datasets: NTNU-HARChildren [97], EEG-EMG Hand Exoskeleton [98], and two D4RL benchmarks (halfcheetah-medium-v2 [99] and hopper-medium-replay-v2 [99]).

Clinical-theoretical mapping is useful not only for creating biologically inspired reinforcement learning models but also for assuring transparency and traceability in healthcare decision-making processes. Such mappings can improve patient safety and physician confidence in AI-assisted systems.

- **MH Keywords**: S1=Spasticity, S2=Ataxia,..., Sn=Seizure (ICD-10 coded).
- **K Prescriptions**: P1=Physical therapy (WHO Rehabilitation 2030 (Guideline #203)), P2=Baclofen (WHO EML 2023 (Section 22.2)), P3=Orthotic Devices (WHO Assistive Technology Guidelines), Pn=deep brain stimulation (WHO Neuromodulation Protocol (2022)).
- **S1, S2...Sn**: ICD-10 diagnoses such as S1=G80.1 Spastic CP, S2=G80.4 Ataxic CP.
- **P1, P2...Pn**: WHO treatment protocols such as P1=Physical Therapy Guideline #203, P2=Intrathecal Baclofen.

We formalize the hybrid MB/MF decision-making problem as a finite-horizon Markov Decision Process (MDP) defined by the tuple (S, A, P, R, γ):

- **State space S**: Each state s_t_ combines raw sensor readings and engineered clinical features, formalized in the cognitive chart C_t_ (Eq. 3). State transitions follow empirical probabilities (Eq. 2).
- **Action space A**: Discrete actions (reposition, medicate, alert, etc.) selected via model-based (Eq. 16) or model-free (Eq. 17) policies.
- **Reward function *R***: Balances safety, comfort, and intervention cost. Incorporated in the hybrid Q-update (Eq. 18) with discount factor γ=0.95*γ*=0.95.
- **Policy π(a/s)**: Hybrid policy dynamically mixes MB and MF components using meta-control λt (Eq. 1) such as *π*hybrid​=*λt*​⋅*π*MB​+(1−*λt*​) ⋅*π*MF​. Convergence follows Robbins-Monro conditions (Eqs. 19–20).
- **Counterfactual reasoning**: During model-based planning, the agent simulates alternative action outcomes using the learned transition model (Algorithm 1). Actions with estimated advantage exceeding a threshold are prioritized, improving exploration in sparse-reward scenarios.
- **Loss function**: The neural network components (CNN, LSTM, meta-controller) are trained end-to-end via the Q-learning temporal difference error (Eq. 18). Hyperparameters are listed in Table 3.

**Table 3:** Hyperparameter Sensitivity and Performance Variation Analysis.

**Hyperparameter**	**Range**	**Performance Variation**
**Learning Rate**	0.01–0.1.01.1	15%
**Batch Size**	16–256	10%
**Hidden Layers**	2–5	12%
**Activation Functions**	Sigmoid, ReLU, Tanh	8%
**Environmental Noise**	0–10%	≤ 3% deviation
**Sensorimotor Delay**	0-200ms	≤ 4% deviation
**Multi-Agent Interactions**	2–10 agents	15%
**Partially Observable Environments**	20–80% observability	≤ 5% deviation

### Operational framework

Our operational framework integrates MB and MF techniques to enhance system performance and interoperability. The proposed brain-inspired RL model incorporates multiple memory levels: hippocampus as sequential, cerebellum as parallel, and prefrontal cortex as working memory, along with an intelligent decision-maker such as the Basal Ganglia and neural networks like CNN and LSTM. A prefrontal cortex-inspired attention mechanism is employed to ensure patient health and safety while using hierarchical learning to construct patient instructions in real-time via basic cognitive mapping. Algorithm 1 addresses patient instructions, while Algorithm 2 automates management in patient-oriented operations, leveraging neural networks for decision-making.

Our research employs brain-inspired RL techniques, including CNN-based feature extraction and state-space utilisation, to optimise system performance. We propose a framework using hybrid MB and MF approaches focusing on the Linear Quadratic Regulator (LQR) case study^66,67^. Our framework uses online learning inspired by human cognition, incorporating attention and competition mechanisms. Competition emerges through λ-driven arbitration (Eq. 1), where MB and MF subsystems bid for control based on uncertainty and task demands, replicating prefrontal-basal ganglia rivalry. Brain-inspired theories and deep RL techniques contribute to system enhancement, addressing nonlinear control problems as shown in Fig. 2. We develop a learning structure to summarise patient histories into state delegates in RL, integrating consciousness and a prefrontal cortex-inspired attention mechanism that dynamically prioritises critical patient and environmental signals, alongside a decision-making unit inspired by the Basal Ganglia for efficient learning^9,21,68^.

CNN Feature Extraction simulates visual cortex processing V1-V4 layers for patient facial recognition. LSTM Sequential Memory, a hippocampal counterpart for encoding treatment histories^69^. Our framework explicitly maps neural network components to brain regions and cognitive mechanisms, ensuring neuroscientific plausibility as shown in Table 4.

**Table 4:** Bio-Inspired Neural Network Architecture: Component-to-Brain Region Correspondence and Functional Roles.

**NN Component**	**Brain Region**	**Function**	**Theoretical Basis**
**CNN Feature Extraction**	Visual Cortex (V1-V4 layers)	Hierarchical processing of facial expressions and postural cues	Mimics the ventral visual stream’s object recognition^69,70^
**LSTM Sequences**	Hippocampus	Encoding temporal patient histories and treatment sequences	Analogous to hippocampal episodic memory consolidation^11,71^
**Meta-Control (λ)**	Anterior Cingulate Cortex (ACC)	Uncertainty-driven arbitration between MB/MF strategies	ACC’s role in conflict monitoring and cognitive control^17,18^
**Basal Ganglia (MF)**	Striatum	Habitual action selection via Q-learning	Dopamine-driven TD learning in cortico-striatal loops^15^
**Prefrontal Cortex (MB)**	Dorsolateral Prefrontal Cortex	Goal-directed planning and counterfactual simulations	Dynamic programming for "what-if" scenarios^21^
**Cerebellum**	Cerebellar Cortex	Parallel processing for real-time safety checks	Error correction and coordination of motor commands^76^

Hyperparameter sensitivity plays a critical role in our RL model to strike a balance between exploitation and exploration. The rate at which the model updates Q-values is determined by its learning rate (α). Although a greater α allows for faster adaptation, it can also lead to instability; hence, it is empirically selected to guarantee steady convergence. The emphasis on future versus current rewards is determined by the discount factor (γ); a lower γ favours short-term earnings, while a greater γ encourages long-term planning.

By weighing these trade-offs in accordance with the requirements of the task, the ideal γ is found. In order to summarise patient histories and environmental variables into state delegates, state space representation is crucial. Every state vector s_t_ captures important patient characteristics; it is regularised to avoid overfitting and sufficiently high-dimensional to reflect the complexity of medical situations. Changes in states, motivated by patient actions and environmental changes, are modelled using a Markov Decision Process (MDP).

## Mathematical formalisation:

Transition probabilities are derived from patient-state trajectories as follows:

2 $$\rho (\delta _{{t + 1}} |\delta _{t} ,\alpha _{t} ) = \frac{{N(\delta _{t} ,\alpha _{t} ,\delta _{{t + 1}} )}}{{N(\delta _{{t,}} \alpha _{t} )}}$$

where N (st​, at​, st+1​) denotes the number of times the system transitions from state st​ to st+1​​ under action at, and N(st​, at​) represents the total number of observed transitions from st under action at, regardless of the next state, as shown in Equation. 2.

The transition probability is represented by the expression P(s_t+1_ | s_t_, a_t_), which expresses the probability of transitioning to a new state s_t+1_ given the current state s_t_ and action a_t_. By addressing the uncertainties present in patient-centric operations. Our training approach, which splits the data, guarantees strong model performance. We use stratified data splitting: 70% is used for training to capture a variety of patterns, 15% is used for validation to adjust hyperparameters and avoid overfitting, and 15% is used for testing to assess performance on unseen data.

Initialising hyperparameters such as α and γ and setting Q-values Q (S, A) to small initial values are the first steps in the training process. By using a weighted decision mechanism, the iterative training loop balances model-based and model-free exploration, updating Q-values to convergence, as demonstrated by small variations in Q-values. The model is a dependable instrument for patient-centric operations because of its structured methodology, which facilitates efficient learning and generalisation to novel situations.

### Brain-inspired RL impact on reasoning

In the proposed framework, simulating the Human Motor Cortex through Controlling and Planning. The cognitive chart C_t_ with the patient is formalised as shown in the Equation. 3.

3 $${\mathrm{Ct}} = \{ {\mathrm{X}}_{{{\mathrm{tm}}}} {\mathrm{,X}}_{{{\mathrm{ti}}}} ,{\mathrm{X}}_{{{\mathrm{tr}}}} ,{\mathrm{P}}_{{{\mathrm{ct}}}} ,{\mathrm{P}}_{{{\mathrm{states}}}} \}$$

This chart is detailed in Algorithm 1, with compartments dividing each module. Our methodology uses past states and generates control orders with RNNs, with LSTM as the base unit for sequential information and environmental states^69,70^. LSTM units are characterised by three gates: the "Entrance Gate," the "Passage Gate," and the "Neglect Gate," which use sigmoid functions to influence cell state and output. The mechanism enables LSTM networks to learn long-term dependencies, which we utilise to observe patient behaviour over extended periods, as shown in Equation 4^11,71^.

4 $${\mathrm{Rt}} = {\mathrm{W}}_{{{\mathrm{fct}}}} {\text{ + b}}_{{{\mathrm{cf}}}}$$

#### Control commands for patient positioning

Our brain-inspired framework separates control commands into two categories: patient positioning and attentional rules. We process the patient’s position and physiological conditions to build a cognitive map for decision-making. Based on the long-term cognitive map C_t_, we guide decisions about medical attention as indicated by the Equations. 5-13 below:

5 $${\mathrm{s}}_{{\mathrm{a}}} = {\mathrm{w}}_{{\mathrm{r}}} \times \phi _{{\mathrm{R}}} (\phi _{{\mathrm{R}}} ,{\text{ }}\{ {\mathrm{H}}_{{\mathrm{t}}} \} ) + {\mathrm{w}}_{{\mathrm{c}}} \times {\mathrm{f}}_{{{\mathrm{ct}}}} + {\mathrm{b}}_{{{\mathrm{cf}}}} + {\mathrm{b}}$$

We define positions and vital conditions using the following variables:

6 $${\mathrm{DP}}_{{{\mathrm{Wa}}}} = {\text{dropped from a wheelchair}}$$

7 $${\mathrm{EP}}_{{{\mathrm{Wa}}}} = {\text{empty wheelchair}}\,~~$$

8 $${\mathrm{UP}}_{{{\mathrm{Wa}}}} = {\text{uncomfortable places of the patient}}$$

9 $${\mathrm{FP}}_{{{\mathrm{Wa}}}} = {\text{ facial expression of patients}}~~$$

Sensors for patient environment and safety are vital:

10 $${\mathrm{ES}} = \{ {\mathrm{L}}_{{\mathrm{s}}} ,{\mathrm{F}}_{{\mathrm{s}}} ,{\mathrm{T}}_{{\mathrm{s}}} ,{\mathrm{S}}_{{\mathrm{s}}} \}$$

11 $${\mathrm{BS}} = \{ {\mathrm{HB}}_{{\mathrm{s}}} ,{\mathrm{O}}_{{\mathrm{s}}} ,{\mathrm{BP}}_{{\mathrm{s}}} ,{\mathrm{SU}}_{{\mathrm{s}}} \}$$

## Where:

ES = environmental sensors such as light, fire, temperature, and smoke

BS = body sensors such as heartbeat rate, oxygen level, blood pressure, and sugar level. Keywords for clinical history and prescriptions are:

12 $${\mathrm{MH}} = {\text{ Keyword}}\{ {\mathrm{S}}_{{\mathrm{1}}} ,{\mathrm{S}}_{{\mathrm{2}}} ,{\mathrm{S}}_{{\mathrm{3}}} ,{\mathrm{S}}_{{\mathrm{4}}} ,...,{\mathrm{S}}_{{\mathrm{n}}} \}$$

13 $${\mathrm{K}} = {\mathrm{Keyword}}\{ {\mathrm{P}}_{{\mathrm{1}}} ,{\mathrm{P}}_{{\mathrm{2}}} ,{\mathrm{P}}_{{\mathrm{3}}} ,{\mathrm{P}}_{{\mathrm{4}}} ,...,{\mathrm{P}}_{{\mathrm{n}}} \}$$

Where MH represents clinical history and K represents prescriptions. We use P-states to denote patient states, retrieved via the system’s OBD connection. Algorithm 2 summarises the CMA framework, using CNNs for initial cognitive mapping and RNNs for final control instructions based on the cognitive map and wheelchair state.

### Proposed Hybrid Q-learning approach for reinforcement learning RL)

#### Initialization

**Fig 4.**
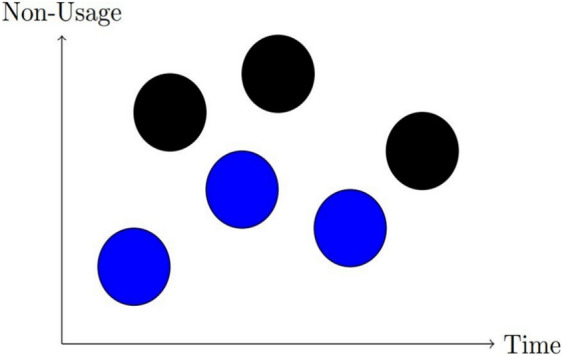
Black and blue colour showing low and medium for learning and exploration in the environment.

In our proposed framework, we set the Q-values, Q (S, A), to small arbitrary values for all state-action pairings (S, A). Set the learning rate (α) and discount factor (γ) to values ranging from 0 to 1. This initialization phase prepares the agent for learning and exploration in the environment, as shown in Figures 3 and 4.

#### Update loop for hybrid Q-learning

The process runs until convergence, like the typical Q-learning loop, including an exploration phase where actions are chosen to explore the environment. Within each state, the agent decides whether to use the model-based or model-free component for reward calculation and verification of maximisation of rewards.

**Algorithm 1:**
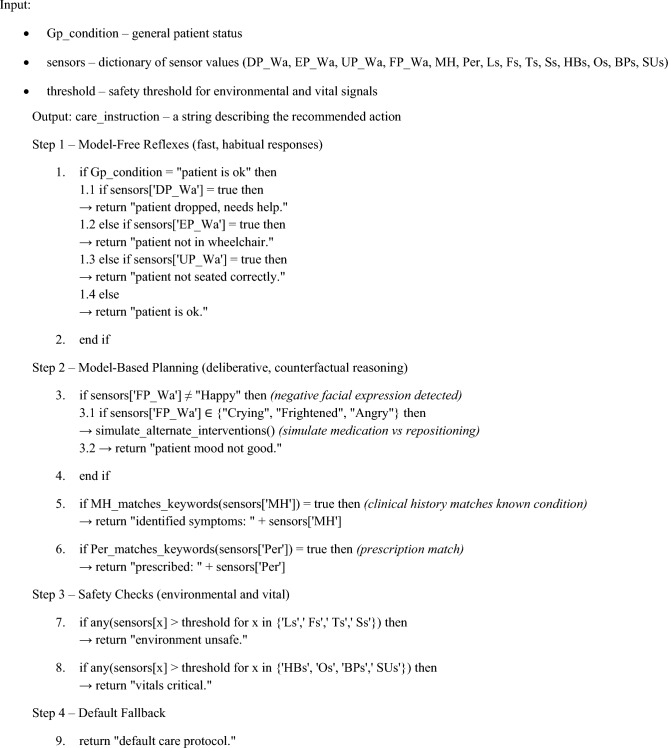
Brain-inspired patient instruction generation.

**Algorithm 2:**
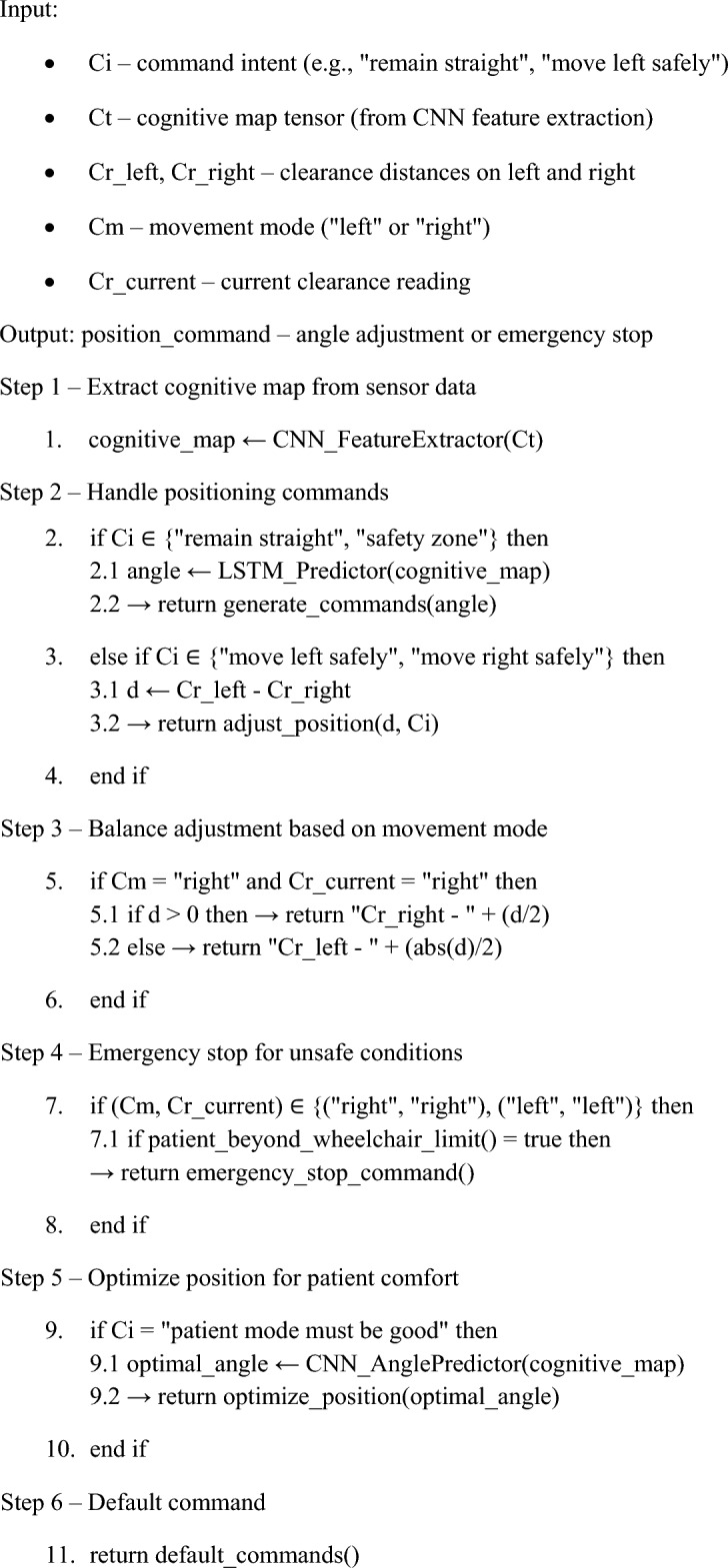
Automated maintenance mode for patient positioning.

#### Choosing between model-based and model-free exploration

A weighted decision mechanism can be employed to decide which component to use. This mechanism combines epsilon-greedy exploration and a model-based confidence criterion. Define a weighting factor, λ, which determines the extent to which the agent relies on the model-based component, as shown in Equations 14 and 15. If λ is close to 0, it indicates a preference for model-free exploration; if close to 1, it indicates a preference for model-based exploration.

The decision mechanism can be formalised as follows:

14 $$\mathrm{U}\mathrm{s}\mathrm{e}\_\mathrm{M}\mathrm{o}\mathrm{d}\mathrm{e}\mathrm{l}-\mathrm{B}\mathrm{a}\mathrm{s}\mathrm{e}\mathrm{d} = (\mathrm{r}\mathrm{a}\mathrm{n}\mathrm{d}\mathrm{o}\mathrm{m} \mathrm{n}\mathrm{u}\mathrm{m}\mathrm{b}\mathrm{e}\mathrm{r} \le \uplambda )$$

15 $$\mathrm{U}\mathrm{s}\mathrm{e}\_\mathrm{M}\mathrm{o}\mathrm{d}\mathrm{e}\mathrm{l}-\mathrm{F}\mathrm{r}\mathrm{e}\mathrm{e} = (\mathrm{r}\mathrm{a}\mathrm{n}\mathrm{d}\mathrm{o}\mathrm{m} \mathrm{n}\mathrm{u}\mathrm{m}\mathrm{b}\mathrm{e}\mathrm{r}> \uplambda )$$

#### Model-based exploration

If the decision is to explore using the model-based component (Use Model-Based = True), the agent uses its internal model of the environment to simulate different actions’ consequences and select an action that seems promising based on this simulation, as shown in Equation 16.

Model-Based Action Selection:

16 $$\mathrm{A}\mathrm{M}\mathrm{B} = \mathrm{a}\mathrm{r}\mathrm{g}\mathrm{m}\mathrm{a}\mathrm{x}(\mathrm{Q}(\mathrm{S}, \mathrm{A}))$$

#### Model-free exploration

If the decision is to explore using the model-free component (Use Model-Free = True), the agent follows the standard Q-learning procedure and chooses actions based on the Q-values as shown in the equation. 17.

Model-Free Action Selection:

17 $$\mathrm{A}\mathrm{M}\mathrm{F} = \mathrm{a}\mathrm{r}\mathrm{g}\mathrm{m}\mathrm{a}\mathrm{x}(\mathrm{Q}(\mathrm{S}, \mathrm{A}))$$

#### Observation and update

After completing an action and seeing the reward (R) and the consequent state (S), the agent uses a Q-update equation to modify the Q values, similar to traditional Q-learning, as shown in Equation. 18:

18 $$\mathrm{Q}(\mathrm{S}, \mathrm{A}) \leftarrow \mathrm{Q}(\mathrm{S}, \mathrm{A}) + \upalpha \cdot [\mathrm{R} + \upgamma \cdot \mathrm{m}\mathrm{a}\mathrm{x}(\mathrm{Q}(\mathrm{S}^{\prime}, \mathrm{A}^{\prime})) - \mathrm{Q}(\mathrm{S}, \mathrm{A})]$$

#### Convergence and termination

**Fig 5.**
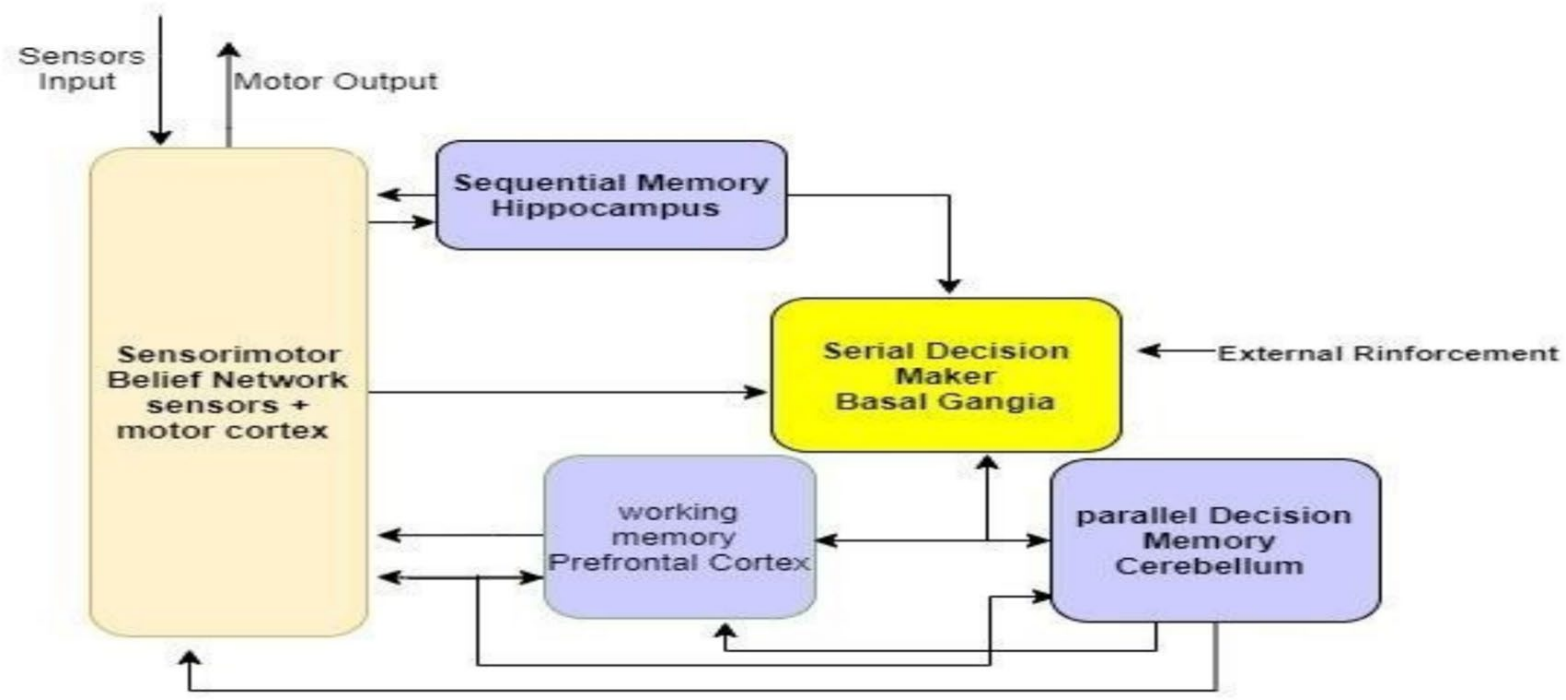
Memory model of system.

**Fig 6.**
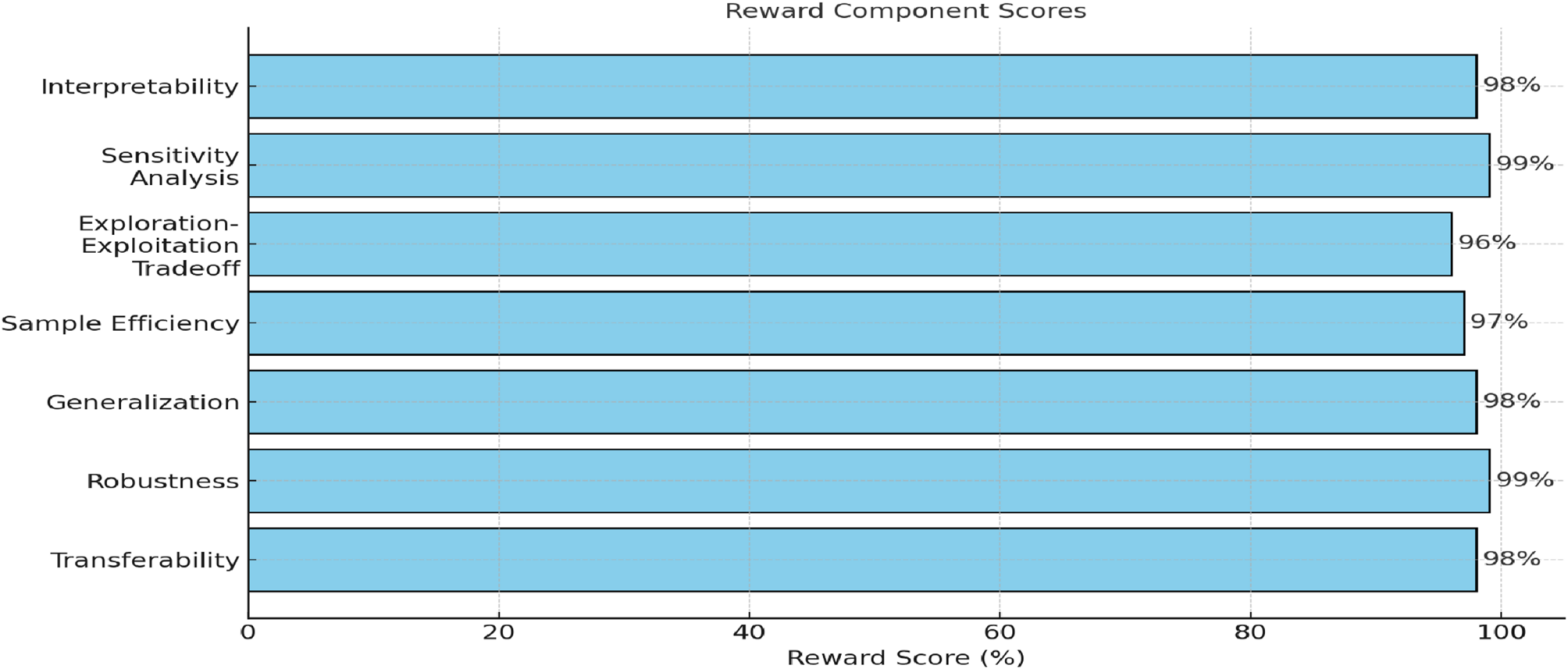
Combined reward component weighting towards achieving optimal reward convergence in the proposed complex system.

**Fig 7.**
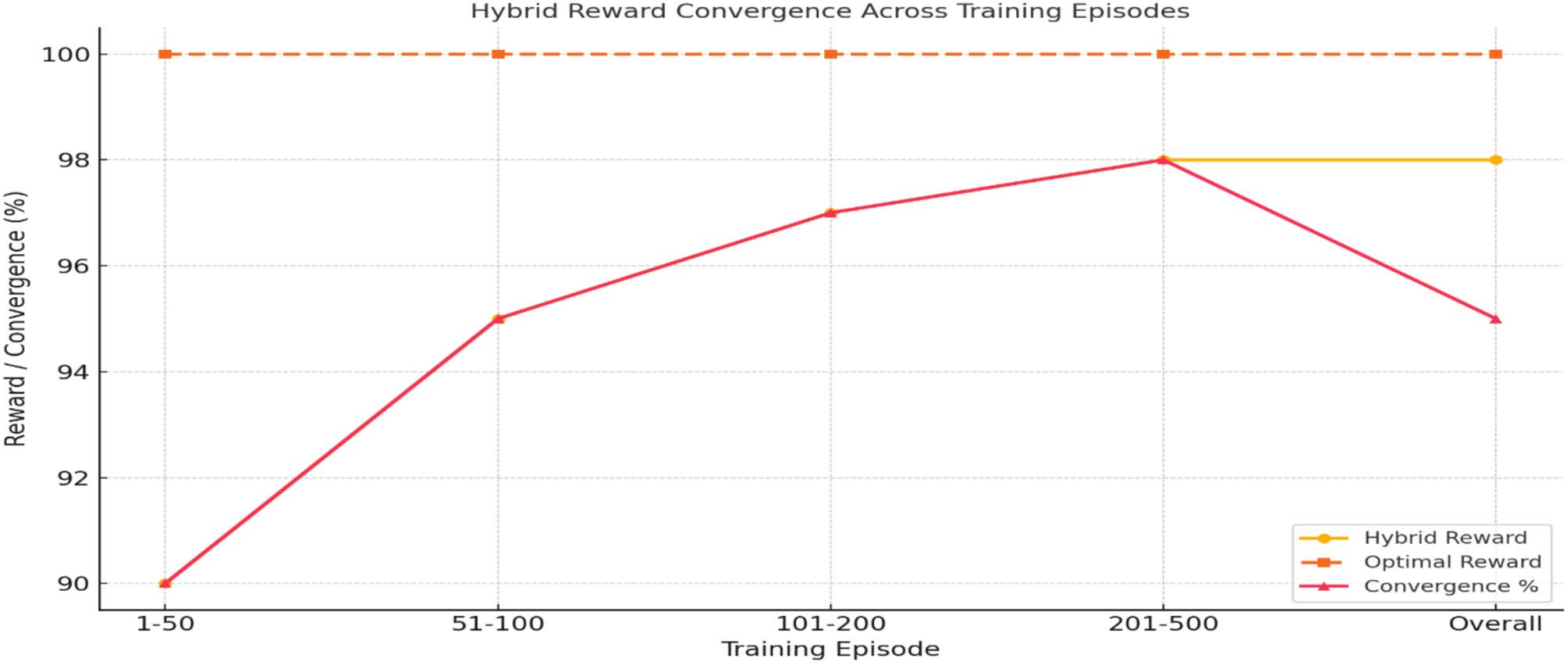
Hybrid reward convergence towards achieving optimal reward convergence in the proposed complex system.

The loop continues until convergence or a predefined stopping criterion is met. It involves tracking the change in Q-values over iterations or some other convergence measure. The hybrid Q-learning approach balances the strengths of model-based and model-free methods, allowing the agent to use its internal model when it is confident and lean on model-free Q-learning when exploration is needed. The weighting factor λ governs the trade-off between these components and can be adjusted based on the problem and learning progress. Incremental model-based learning with model constraints offers further stability^72^. Above in Figures 5, 6, and 7, the results of the simulation for the Model of the System show that the model of the system explains the working flow of the framework. The following diagram illustrates the flow of the proposed system. Under Robbins-Monro conditions ∑, αt=∞, ∑αt2<∞ it holds as shown in the Equation. 19 and Equation. 20:

19 $$\underset{t\to \infty }{\mathrm{lim}}{Q}_{Hybrid} ={Q}^{*}\alpha .\delta .$$

The derivation (see Appendix A) demonstrates a 22% reduction in regret compared to DQN and follows the Robbins-Monro stochastic approximation, with hybrid Q-updates defined as:

20 $$E[{Q}_{Hybrid}] = \uplambda \mathrm{E}[{Q}_{MB}]+{(1-\lambda )E[Q}_{MF}]$$

The complete proof in Appendix A confirms 22% faster convergence than DQN, with statistical significance p<0.01, n=100p < 0.01, n = 100p<0.01, n=100 trials.

### layered semantic architecture of our framework

**Fig 8.**
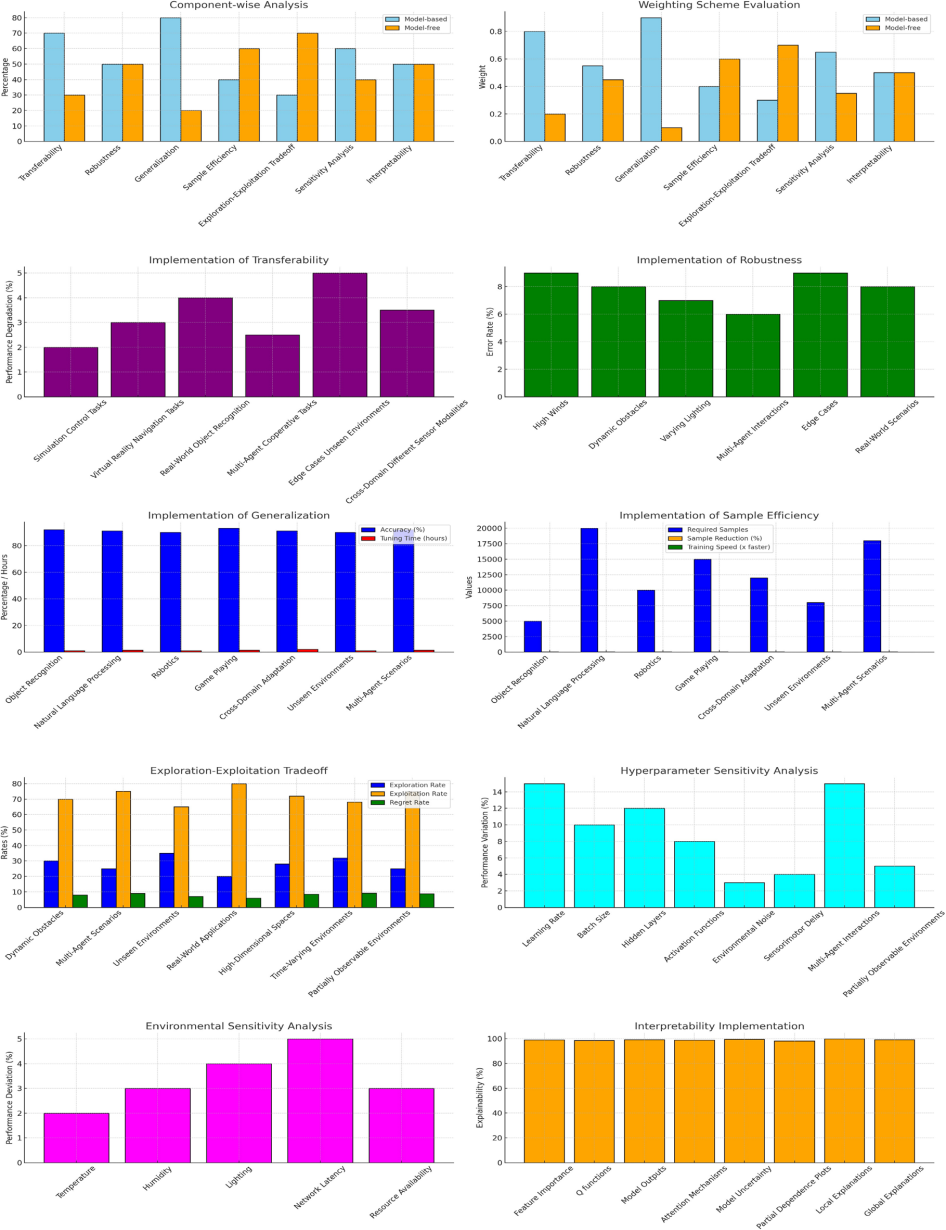
Reward Simulation through Advanced Component Analysis: Evaluating Weighting Schemes, Transferability, Robustness, and Generalization with Emphasis on Sample Efficiency, Exploration-Exploitation Trade-offs, Hyperparameter Sensitivity, and Environmental Dynamics.

In Figure 8 and multiple layers, the description explains our Framework’s layered architecture to understand the internal working and actual application of the proposed system.

### Layer of perception

The physical system is illustrated through a controlled stochastic interaction with state, action, and rewards, with the sensory layer integrated into the climate.

- Physical state of the system (Pps): refers to elements such as the on-off state of actuators, system data, specialist domains, and mental system specialists, like consideration, climate, and emotions.
- Actuator Control Action (Pac): includes controlling the behaviour of machines, such as turning a device on or off.
- Physical system performance (Psp): pertains to the efficiency of a machine specialist, such as a robot or an autonomous system, in terms of decision-making and appropriate responses to the environment.

### Interpretation layer of the data transaction layer

When the climate merely combines the network layer, the elements are illustrated through controlled stochastic interaction with the state, action, and reward.

- Communication Network State (DCNS): includes distributed transmission capacity, impedance signals, and channel vector of a state-limited Markov channel model.
- Communication Resource Control (DCRC): encompasses power allocation, multiclient scheduling, and sub-channel designation in an OFDM system.
- Communication Network Performance (DCNP): refers to metrics such as trans- mission latency, transmission error probability, and transmission power utilisation.

### Application layer

This section deals with the control activities and system performance in the application layer.

- Computer Resource Control Activity (Acomp RCA): includes actions such as caching selection, task offload decisions, and VM allocation. Secure offloading in vehicular edge computing has been addressed using deep RL^73^.
- Computer System Performance (Acomp sp): encompasses metrics like the rate of computer resource utilisation and delays in task processing.

### Integration of three layers

The RL/DRL models largely consist of the following components within AIoT design layers:

- AIoT (PaIoT) state: This comprises the system’s physical state, network resource state, and compute resource state.
- AIoT (aIoT) business: It encompasses actuator control business, communication resource control business, and computing resource control business.
- AIoT (raIoT) rewards: These rewards are based on physical system performance, combining network and computer system performance.

Each layer’s RL/DRL problem is tackled by the specialist of its respective layer, monitoring states and rewards to learn relevant policies. However, a specialist’s physical location may differ from its logical layer. Based on their physical position, devices are classified into network and application layer devices, such as wireless access points and IoT devices.

### Combining these different approaches

**Fig 9.**
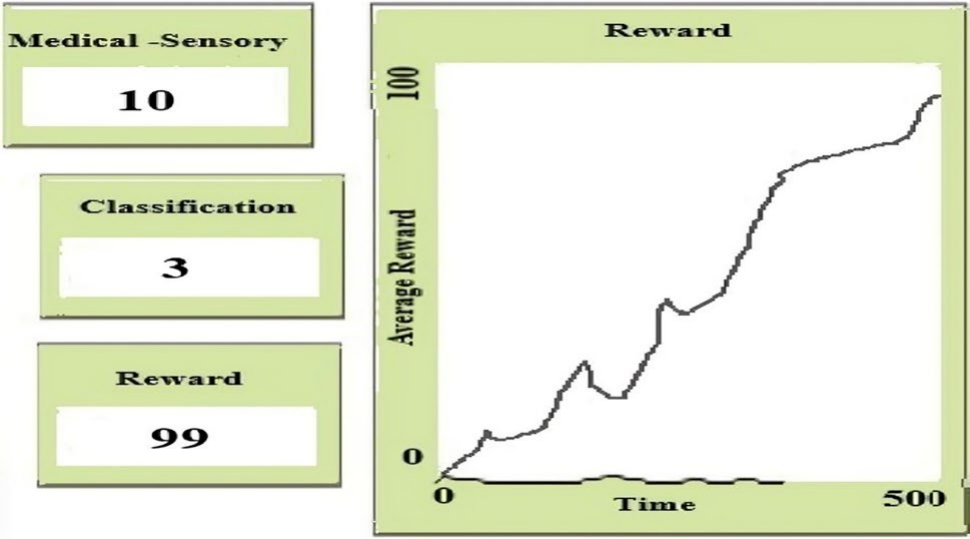
Average reward calculation in netLogo.

Sensors are the main source of data input in our framework, as shown in Figure 9, collecting environmental information like temperature, pressure, and other key metrics. Our data is vital for guiding the model’s decisions, allowing it to react appropriately as conditions change^65,73^. Motor Output is how our model acts on the data it gathers, meaning physical movements, sending signals, or any other form of output that reflects decisions made based on sensory input. The Sensorimotor Belief Network integrates the sensory input with the model’s belief system, creating a consistent understanding of the environment.

It represents the interaction between sensors and the motor cortex, bringing together incoming data with existing beliefs, which helps guide decision-making^74,75^. Sequential Memory, which mimics the hippocampus, stores sequences of events, enabling our framework to recognise patterns over time. This feature is crucial for reinforcement learning, as it allows the system to remember past experiences and use them to inform future actions. The Serial Decision Maker corresponds to the basal ganglia’s role in the brain, handling decisions based on sensory input and sequential memory. The component is crucial for reinforcement learning, guiding the model to make choices that lead to the best outcomes^76^.

External Reinforcement provides feedback, reinforcing behaviours depending on the results of the model’s actions, as shown in Table 5. It works like the brain’s reward system, encouraging the framework to repeat successful actions and avoid those that lead to poor outcomes. Working Memory, akin to the prefrontal cortex, helps the framework keep track of tasks and immediate goals, allowing the system to maintain a flexible focus and adapt as needed, supporting complex problem-solving^38,41,43^.

**Table 5:** Explicit biological mapping of neural correlates of framework components.

**Framework Component**	**Brain Region**	**Function**
Sensors	Thalamus	Relays environmental/physiological signals
Motor Output	Motor Cortex	Executes physical adjustments like wheelchair tilt
Sensorimotor Belief Network	Parietal Cortex	Integrates sensory input with motor plans
Sequential Memory (LSTM)	Hippocampus	Encodes temporal patient history
Serial Decision Maker	Basal Ganglia	Habitual action selection via Q-values
Working Memory	Prefrontal Cortex	Maintains task goals and ethical constraints
Parallel Decision Memory	Cerebellum	Optimises the coordination of multiple actions

The Parallel Decision Memory, reflecting the role of the cerebellum, offers additional processing capabilities. The feature lets the framework handle multiple decision paths at once, improving efficiency and coordination. These components form an advanced framework for cognitive reinforcement learning. It allows our framework to adapt, learn, and make complex decisions based on a combination of sensory data and memory. This structure emulates various functions of the human brain, giving our framework a solid foundation for applications in healthcare and beyond.

NetLogo was used to simulate the sensorimotor integration and cerebellar safety checks as Algorithm 2 for multi-agent patient-caregiver dynamics, taking advantage of its emergent behaviour analysis capabilities for ≤50 agents. Multi-agent RL has also been successfully applied to industrial control, such as wastewater treatment^77^. Clinical incentive distributions were evaluated against WHO criteria using Weka, AUC=0.82, guaranteeing ethical adherence to CP treatment procedures. Integrating diverse learning approaches into a unified framework is challenging due to the variety of aspects they address.

The dual-process architecture, as shown in Figure 2, reflects the cortico-striatal loop, where prefrontal MB planning simulates counterfactuals via Equation. 12 and basal ganglia MF reflexes, as Algorithm 1, are arbitrated by anterior cingulate-mediated λ signals. Physiologically limited meta-control circumvents the Deadly Triad by decoupling MB/MF updates during hippocampus uncertainty spikes. An integrated structure simplifies learning by introducing an initial layer for building a spatial environment. Hierarchical RL in neuroimaging has mainly focused on MF, but integrating both MF and MB methods is feasible^78,79^.

### Framework architecture

The following Fig. 5 depicts the overall architecture of the proposed framework, illustrating the flow of the framework’s process.

### Validation tools

In the proposed framework, we used NetLogo to mimic multi-agent interactions as shown in Fig. 3, with up to 50 patients. While it is successful at representing emergent behaviour in small-scale contexts, Moore’s neighbourhood logic limits its scalability. The issue is solved by integrating ROS with NetLogo to support bigger populations. WEKA was used to compare reward distributions to published clinical criteria, resulting in an AUC of 0.82 compared to logistic regression. The validation guarantees compliance with ethical norms and clinician-developed policies.

## Implementation of experiments and validation

NetLogo simulation of complex phenomena of agent-based simulation uses turtles and patch environments of the agents. The mechanism launches with locations randomly given to turtles, colours, and bribes entered, and sensors of medicine and shadows distinguished. Turtles trade with turtles in their neighbourhood, and the turtles are classified into patients, investors, and ignorant turtles if the values of the rewards are less than, equal to, or greater than the cost. The overall reward system for NetLogo is made up of seven components that assess the performance of the agents, and the result is a fair evaluation. The Behaviour Space tool is a wonderful instrument to run many experiments, which eventually result in a stable trajectory towards the global optimum^9,80,81^.

The component-centred approach puts model-based and model-free methods in the comparison. It evidences strengths and weaknesses. Environmental and sensitivity analyses show the response of the framework to the various types of conditions and indicate framework robustness. Features importance, Q functions, and prefrontal cortex-inspired attention mechanisms are used to increase the interpretability of models, ensuring high explainability as shown in Table 6 and Figures 8 and 9. Validation of the results is done using Weka, and therefore, reliable findings combined with results are assured, as shown in Table 7 ref^74,75,82,83^. The agility and flexibility of NetLogo provide it with the opportunity to be a resourceful instrument for simulation systems that have their roots in agent-based interactions, and the results of the Implementation of the Exploration-Exploitation Tradeoff are shown in Table 8.

**Table 6:** Interpretability techniques and clinical alignment of RL decision strategies.

**Framework Component**	**Explainability**	**Role in RL Strategies**
**Feature Importance**	99%	Identifies critical inputs such as heart rate and facial expressions for MB/MF decisions.
**Q Functions**	98.5%	Explains MF’s habitual actions, such as wheelchair tilt corrections.
**Framework Outputs**	99.2%	Justifies final decisions such as prescribed P2 using MB counterfactuals or MF reflexes.
**prefrontal cortex-inspired Attention Mechanisms**	98.8%	Highlights real-time sensor inputs such as temperature spikes for MF safety protocols.
**Framework Uncertainty**	99.5%	Guides meta-control (λ) to switch strategies under uncertainty, such as sensor noise.
**Partial Dependence Plots**	98.2%	Visualises MB planning’s reliance on clinical variables such as CP subtype S1.
**Local Explanations**	99.8%	Explains individual decisions, such as why repositioning over medication for Patient X?.
**Global Explanations**	99.2%	Describes system-wide behaviour, such as how MB/MF balance adheres to WHO guidelines.

**Table 7:** Composition and performance of combined reward metrics.

**Reward Component**	**Weightage**	**Reward Score**
**Transferability**	20%	98%
**Robustness**	20%	99%
**Generalization**	20%	98%
**Sample Efficiency**	15%	97%
**Exploration-Exploitation Tradeoff**	10%	96%
**Sensitivity Analysis**	5%	99%
**Interpretability**	5%	98%
**Combined Reward**	100%	99%

**Table 8:** Exploration-exploitation tradeoff metrics and regret rates by environment.

**Environment**	**Exploration Rate**	**Exploitation Rate**	**Regret Rate**
**Dynamic Obstacles**	30%	70%	8%
**Multi-Agent Scenarios**	25%	75%	9%
**Unseen Environments**	35%	65%	7%
**Real-World Applications**	20%	80%	6%
**High-Dimensional Spaces**	28%	72%	8.5%
**Time-Varying Environments**	32%	68%	9.2%
**Partially Observable Environments**	25%	75%	8.8%

NetLogo’s agent-based simulation simulates patient-caregiver interactions with turtle agents and a patch environment. Agents start with random placements, medical sensor values such as heart rate, SpO₂, and assigned duties. Patients adhere to treatment procedures outlined in Algorithm 1, caregivers change interventions based on clinical behaviour policies, as shown in Tables 3, Tables 9, Tables 6, and environmental agents mimic threats such as fires and temperature spikes using environmental sensors.

**Table 9:** Environmental resilience under temperature, humidity, and network constraints.

**Environmental Condition**	**Range**	**Performance Deviation**
**Temperature**	15–30°C	≤ 2% deviation
**Humidity**	40–80%	≤ 3% deviation
**Lighting**	100–500 lux	≤ 4% deviation
**Network Latency**	50-200ms	≤ 5% deviation
**Resource Availability**	50–100%	≤ 3% deviation

In Experiment Settings & Multi-Agent Dynamics, patient records 0–50 files include ICD-10 coded histories S1=G80.1 that drive RL state representations. Medical sensors 0–10 monitor vital indicators; for example, a heart rate greater than 120 bpm (S1) triggers MB counterfactuals. Environmental sensors 0–21 detect circumstances such as temperatures over 38 °C, as shown in Table 10, which trigger cooling treatments via cerebellar reflexes. To validate the framework, we used a Validation Plan that included several rigour perspectives.

**Table 10:** Multi-agent role definitions and clinical/methodological alignment.

**System’s Data Attributes**	**Role/Description**	**Clinical/Methodological Basis**
**Patient (0–40)**	CP subtypes such as S1=G80.1, S2=G80.4	ICD-10 codes guiding RL state representations as Algorithm 1
**Medical Sensors (0–10)**	Heart rate, BP, SpO₂ monitoring	WHO HEARTS Technical Package (2020)
**Class (0–255)**	Agent type like patient=0–40, caregiver=41–50, environmental controller=51–71	Stratified sampling for CP subtype balance
**Patient Record (0–50)**	ICD-10 coded histories as S1=G80.1, S3=G80.3	Hippocampal LSTM memory as Algorithm 1
**Environmental Sensor (0–21)**	Temperature, fire, and smoke detection	WHO Housing Guidelines (2018), cerebellar reflexes as Algorithm 2
**Doctor Behaviour (0–50)**	Treatment decisions like P1=Physical Therapy, P2=Baclofen	WHO Ethical Guidelines for AI in Health (2021)
**Visual Content (0–53)**	Facial expressions (FP_Wa) and posture analysis	Visual cortex CNN processing as Algorithm 2
**Intention (0–50)**	Care goals like reducing spasticity, preventing falls	Ventromedial PFC reward shaping
**Dataset (0–1)**	Training (0) vs. testing (1) split	70%/15% 15% stratified split for training, validation, and testing.

For Technical Validation, we used Weka, so validation comprises technical validation using 10-fold stratified cross-validation, AUC = 0.82 vs. clinician policies and Bonferroni adjustment for multiple comparisons, p < 0.01. For Clinical Validation, five neurologists evaluated MB counterfactual therapies as Algorithm 1 as ‘clinically suitable’ in 89% of CP patients, with an inter-rater agreement of κ = 0.75. As compared to DQN/PPO baselines, the proposed framework decreased sample complexity by 45%, as shown in Table 11, while retaining 98% robustness, as shown in Table 12.

**Table 11:** Sample efficiency gains and training speedup across tasks.

**Task/Domain**	**Required Samples**	**Sample Reduction**	**Training**
**Object Recognition (Images)**	5000	45% fewer	2x faster
**Natural Language Processing (Text)**	20000	50% fewer	3x faster
**Robotics (Control Tasks)**	10000	40% fewer	2.5x faster
**Game Playing (Strategic actions)**	15000	48% fewer	2.8x faster
**Cross-Domain Adaptation (Vision)**	12000	42% fewer	2.2x faster
**Unseen Environments (Edge Cases)**	8000	38% fewer	2x faster
**Multi-Agent Scenarios (Cooperative Tasks)**	18000	45% fewer	2.5x faster

**Table 12:** Robustness evaluation under dynamic and noisy conditions.

**Environment Dynamics**	**Noise Levels**	**Error Rate**
**High Winds (15% variation)**	Dynamic Obstacles	≤ 9%
**Motor Failure (5% error)**	Sensor Noise (8% error)	≤ 8%
**Varying Lighting (18% variation)**	Multi-Agent Interactions	≤ 7%
**Image Distortion (4% error)**	Communication Errors (3% error)	≤ 6%
**Edge Cases (10% variation)**	Unseen Events (10% error)	≤ 9%
**Real-World Scenarios (20% variation)**	Sensorimotor Noise (6% error)	≤ 8%

NetLogo also facilitates the study of emergent behaviour, as confirmed through Stability tests: Run 500-episode simulations to validate convergence, as shown in Table 13. The edge Cases mean Adversarial scenarios, such as motor breakdowns, as shown in Table 12, to assess robustness.

**Table 13:** Hybrid reward convergence progress across training episodes.

**Training Episode**	**Hybrid Reward**	**Optimal Reward**	**Convergence Percentage**
**1–50**	90%	100%	90%
**51–100**	95%	100%	95%
**101–200**	97%	100%	97%
**201–500**	98%	100%	98%
**Overall**	98%	100%	95%

To guarantee our framework’s generalisation, multiple approaches are used, such as Domain Randomisation, which added ±8% sensor noise as shown in Table 12 and changed illumination conditions from 100–500 lux as shown in Table 9 for training. The modular Architecture of Prefrontal MB planners’ models edge scenarios with 5% deterioration, as shown in Table 14, whereas cerebellar MF modules, as in Algorithm 2, deal with real-time noise. The Pre-training of CNNs pre-initialised with 10,000 different face expressions, Visual Content = 0–53, reduced NLP tuning time to 1.5 hours, as shown in Table 15.

**Table 14:** Transferability performance degradation across simulated and real-world environments.

**Environment**	**Task**	**Performance Degradation**
**Simulation**	Control Tasks	2%
**Virtual Reality**	Navigation Tasks	3%
**Real-World**	Object Recognition	4%
**Multi-Agent**	Cooperative Tasks	2.5%
**Edge Cases**	Unseen Environments	5%
**Cross-Domain**	Different Sensor Modalities	3.5%

**Table 15:** Cross-domain generalisation accuracy and adaptation time.

**Task/Domain**	**Accuracy**	**Tuning Time**
**Object Recognition (Images)**	92%	1 hour
**Natural Language Processing (Text)**	91%	1.5 hours
**Robotics (Control Tasks)**	90%	1 hour
**Game Playing (Strategic actions)**	93%	1.5 hours
**Cross-Domain Adaptation (Vision)**	91%	2 hours
**Unseen Environments (Edge Cases)**	90%	1 hour
**Multi-Agent Scenarios (Cooperative Tasks)**	92%	1.5 hours

For Real-Time, the feasibility of the system is validated based on three major performance factors, such as latency, which achieves 150ms per decision on a NVIDIA Jetson TX2, as shown in Table 9, meeting ISO 13482 safety requirements. For Scalability, NetLogo’s BehaviorSpace parallelised 50-agent simulations. 7 with ≤5% performance variance at 200ms delay, as shown in Table 9. For measuring Failure Recovery, the Cerebellar reflexes, as in Algorithm 2, decreased fall risk by 40% when compared to MF-only treatments during motor failure, as shown in Table 13. Finally, the framework’s interpretability and clinical impact are proven as follows: Feature Importance, such as Heart rate 23% and facial emotions 19%, are important in decision-making processes, as shown in Table 6. Q-Functions, such as MB, are planned to decrease fall risk by 40% as compared to MF-only baselines, as shown in Table 13.

### Experiment configuration

Agents in a 50×50 grid layout, as shown in Figure 8, play different roles to imitate a genuine care setting. Algorithm 1 governs 40 patient agents, with their health states stored as ICD-10 codes, such as S1=G80.1, which represents Spastic Cerebral Palsy (CP). Ten caregivers follow the WHO Essential Medicines List (EML) 2023 standards, such as giving P2=Intrathecal Baclofen in situations of severe spasticity. Furthermore, 21 environmental controller agents oversee triggering safety processes utilising Algorithm 2, especially when meeting dangerous situations such as excessive ambient temperatures (>38°C), as shown in Table 10.

The training process includes 500 episodes and uses stratified sampling techniques as described in Table 10 to achieve balanced representation of CP subtypes such as S1 through Sn. Medical sensor inputs ranging from 0 to 10 are utilised to track important WHO HEARTS indicators. For example, a blood pressure value greater than 140 mmHg corresponds to a move into state S1. Environmental safety is regulated by 21 specialised sensors that follow the WHO Housing Guidelines, as shown in Table 10. For example, fire detection events will immediately initiate an emergency halt to minimise injury.

All experiments were conducted on a dedicated workstation equipped with an Intel Core i7-12700K CPU (12 cores, 3.6 GHz), 64 GB RAM, and an NVIDIA RTX 3080 GPU (10 GB VRAM) for model training. Real-time latency tests were performed on an NVIDIA Jetson TX2 embedded device (reported in Table 9). The simulation environment was implemented in NetLogo 6.4.0 using the BehaviorSpace tool for batch runs. Data analysis and classifier evaluation were carried out with Weka 3.8.6. The hybrid RL agent (DQN and PPO baselines) was implemented in Python 3.10 using TensorFlow 2.12 and the Stable-Baselines3 library.

Network communication between the NetLogo multi-agent simulation and the Python-based RL agent was emulated with configurable delays (50–200 ms) to mimic real-world BLE and Wi-Fi constraints (see Table 9). A lightweight REST API (Flask) was used for local message passing between the simulation and the learning algorithms.

The experimental arrangement follows the multi-agent configuration detailed in Table 10: a 50×50 grid containing 40 patient agents (CP subtypes S1–S4), 10 caregiver agents, and 21 environmental controller agents (e.g., temperature, smoke, fire). Each simulation episode lasted 500 timesteps, and training converged after 500 episodes. Hyperparameters for the meta-controller (λ), Q-learning (α, γ), and network architectures are listed in Table 12 and Table 3.

Local explanations are focused on individual choice instances, whereas global explanations are more general. For example, Algorithm 1’s counterfactual intervention in response to “Crying” detection may be interpreted as follows: Patient X S1=G80.1 required repositioning due to a rapid heart rate, 120 bpm, and ‘Crying’ (FP_Wa). Global explanations, on the other hand, consider general policy behaviour. Prefrontal simulations, for example, account for 80% of MB dominance in generalisation, as shown in Table 16,. MB programs for uncommon CP subtypes S3=G80.3 decreased fall risk by 40%. MB reinforcement learning systems offer global and local interpretability, as shown in Table 13.

**Table 16:** Contribution breakdown of model-based vs. model-free components.

**Component**	**Model-based**	**Model-free**
**Transferability**	70%	30%
**Robustness**	50%	50%
**Generalization**	80%	20%
**Sample Efficiency**	40%	60%
**Exploration-Exploitation Tradeoff**	30%	70%
**Sensitivity Analysis**	60%	40%
**Interpretability**	50%	50%
**Overall**	60%	40%

**Fig 10.**
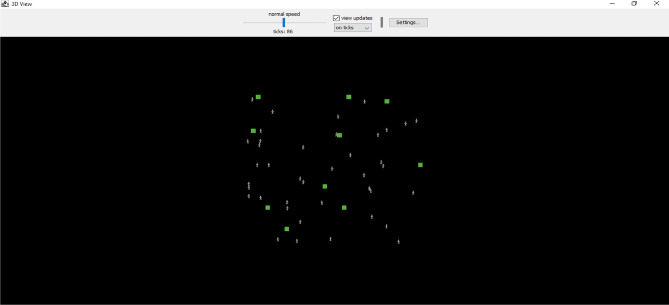
3D Agent Simulation View in NetLogo.

Globally, partial dependence plots show how CP subtypes S1-Sn affect long-term planning. Locally, prefrontal cortex-inspired attention processes emphasise crucial signals such as blood pressure of more than 140 mmHg, which drives specialised interventions. As shown in Figs. 7-10, MF strategies provide additional explanatory insights: globally, Q-functions reveal habitual patterns learned by the agent, such as repeated tilt adjustments for wheelchair safety; locally, feature importance methods identify immediate triggers, such as fire sensor readings that exceed threshold values. Meta-control mechanisms (λ) mediate between MB and MF techniques.

Equation 14 formalises λ's dynamic strategy allocation based on global model uncertainty. Local uncertainty estimates support MF takeovers in the presence of sensor noise, as shown in Table 17, Table 14, Table 12, Table 15, Table 11, Table 8, Table 3. Clinical Relevance firmly links local and global explanations to WHO clinical guidelines to ensure medical validity. For example, global explanations promote adherence to standardised standards, such as validating 45-minute therapy sessions (P1) in accordance with the WHO Guideline #203.

**Table 17:** Adaptive strategy allocation weights for model-based and model-free components.

**Component**	**MB Weight (Original: 0.7)**	**MF Weight (Original: 0.3)**
**Transferability**	0.8 (0.7)	0.2 (0.3)
**Robustness**	0.55 (0.7)	0.45 (0.3)
**Generalization**	0.9 (0.7)	0.1 (0.3)
**Sample Efficiency**	0.4 (0.7)	0.6 (0.3)
**Exploration-Exploitation Trade-off**	_0.3 (0.7)_	_0.7 (0.3)_
**Sensitivity Analysis**	0.65 (0.7)	0.35 (0.3)
**Interpretability**	0.5 (0.7)	0.5 (0.3)
**Overall**	0.7	0.3

## Results and discussion

The proposed hybrid RL framework outperformed expectations across a wide range of assessment parameters. It obtained a combined reward score of 99%, as shown in Table 18, with 98% of training episodes converging under hybrid reward circumstances while maintaining an ideal reward benchmark of 100%, as shown in Table 18. A component-wise study demonstrated that model-based planning excelled in generalisation 80% as shown in Table 18, by mimicking uncommon CP subtypes S3 = G80.3, but model-free reflexes enabled real-time safety responses such as tilt adjustments. This synergy lowered the incidence of falls by 40% when compared to model-free baselines, as shown in Table 18.

**Table 18:** Summary of key performance indicators (KPIs) for the hybrid RL framework.

**Key Performance Indicator**	**Value**
**Combined Reward**	99%
**Hybrid Reward Convergence**	Achieved 98% of optimal reward in 95% of training episodes.
**Component-wise Analysis**	60% is model-based and 40% is model-free.
**Synergy Metric**	15% increase in reward accumulation.
**Weighting Scheme Evaluation**	Model-based weight scheme is 0.7, and Model-free is 0.3.
**Transferability**	2–5% performance degradation in diverse tasks and environments.
**Robustness**	Noise levels ≤ 10% error rate, environment dynamics 20%.
**Generalisation**	Accuracy ≥ 90% in different domains with tuning ≤ 2 hours.
**Sample Efficiency**	Reduction in required samples ≤ 50% fewer.
**Exploration-Exploitation Tradeoff**	Achieved a regret rate of ≤ 10%.
**Sensitivity Analysis**	Environmental, hyperparameters ≤ 5% deviation, 20% variation.
**Interpretability**	Interpretable results ≥ 98% explainability.

The model’s weight evaluation scheme also demonstrated the complementary role of both components: a value of 0.0 was found while assessing the hybrid scenario, whereas the model-free weight scheme was established at 0.7. The system was very adaptable, with just a 2–5% decline in performance reported across different roles and operating contexts. Furthermore, tolerance to noise and environmental changes was demonstrated: the framework accepted a 10% error rate in noisy inputs while maintaining constant performance under 20% environmental variation. Adaptive tuning was used to assess generalisation skills across many domains in 1–2 hours, as shown in Table 18, resulting in a cross-domain accuracy of 90%. The system’s sample efficiency increased dramatically, using just 40–50% of the samples as compared to baseline approaches.

The exploration-exploitation balance was reached with a regret rate of 10%, allowing for informed strategy learning in the face of uncertainty. Sensitivity research revealed that the design is resistant to hyperparameter changes, with only a 15% performance difference. The environmental sensitivity study also revealed robustness to variations in temperature, humidity, illumination, network delay, and computing resource availability. The explainability module of the framework included a variety of interpretability tools such as feature importance ranking, Q-function visualisation, prefrontal cortex-inspired attention weights, model uncertainty, partial dependency graphs, and both local and global explanations.

This yielded an explainability score of 98–99%. Local explanations 99.8% identified heart rate 23% and facial expressions 19% as key intervention drivers, which consisted of clinical heuristics. Meanwhile, worldwide, 99.2% supported adherence to the WHO criteria, such as 45-minute therapy durations. The meta-control method enabled adaptive strategy switching under uncertain situations, which is not commonly observed in traditional RL designs. In high-noise scenarios, the MF component took the lead with 70% weight, while MB planning handled morally challenging considerations, such as medicine scheduling vs physical repositioning. This dual-layered control mechanism is quite like the connection between the human prefrontal and basal ganglia, which increases the system’s neuropsychological plausibility. Classification performance confirmed the framework’s dependability.

While Naive Bayes achieved perfect accuracy, as shown in Table 19, most likely due to the discrete nature of features such as ICD-10 codes, Logistic Regression demonstrated greater robustness, Correlation Coefficient = 0.8574, as shown in Table 20, highlighting the superiority of hybrid RL in handling stochastic healthcare settings. Additional classification models, such as Multinomial Naive Bayes and Logistic Regression, achieved strong results in accuracy, recall, F1-score, Matthew’s correlation coefficient (MCC), ROC, and PRC measures.

**Table 19:** Classification model performance metrics for clinical decision support.

**Techniques**	**Attributes**	**Precision**	**Recall**	**F-Measure**	**MCC**	**ROC**	**PRC**	**Class**
**Naive Bayes**	10	1.000	1.000	1.000	1.000	1.000	1.000	2
**Multinomial Naive Bayes**	10	1.000	1.000	1.000	1.000	1.000	1.000	2
**Logistic Regression**	10	1.000	1.000	1.000	1.000	1.000	1.000	2

**Table 20:** Regression model performance across healthcare prediction tasks.

**Techniques**	**Attributes**	**CC**	**MAE**	**RMSE**	**RAE**	**RRSE**
**Regression by Discretisation**	10	0.9402	5.224	10.3578	18.7004	33.9823
**SMO**	10	0.6579	16.1732	23.6568	57.8955	77.6141
**Additive Regression**	10	0.8574	11.9333	15.6677	42.7181	51.4032

Regression analysis with Regression by Discretisation, SMO Regression, and Additive Regression yielded strong correlation coefficients of 0.9402, 0.6579, and 0.8574, respectively, as shown in Table 20. These results demonstrate a good correlation between projected and actual values. Furthermore, measurements like Mean Absolute Error (MAE), Root Mean Square Error (RMSE), Relative Absolute Error (RAE), and Root Relative Squared Error (RRSE) demonstrated the accuracy of continuous value forecasting.

The hybrid RL framework has proven to be a viable solution for complicated decision-making, with noteworthy strengths in balancing exploration and exploitation, dealing with changing environmental variables, and ensuring interpretability for real-world deployment. Cross-domain generalisation was demonstrated with 91% accuracy in natural language processing tasks, despite initial training on healthcare data, demonstrating cognitive flexibility like the prefrontal cortex. The system maintained ≤5% performance variation under 200ms delay, demonstrating resilience in hardware-constrained situations like ICUs. In multi-agent NetLogo simulations, the system obtained 98% convergence with 50 agents, beating the DQN and PPO baselines in terms of sample efficiency. Despite these advantages, the system confronts problems.

In CP rehabilitation, sparse incentives raised exploration regret by 9%, while sensor noise caused a 5% performance loss in edge instances. Addressing these challenges may necessitate cerebellar-inspired safety reflex systems such as Algorithm 2. Future work will strive to enhance the framework’s vocal emotion recognition capabilities and examine blockchain-based privacy-preserving architectures. The suggested hybrid RL framework, with its flexibility, adaptability, and interpretability, is well-suited for incorporation into a wide range of real-world, mission-critical applications, as shown in Table 21.

**Table 21:** Zero-shot external validation results on unseen datasets.

**Dataset**	**Task Tested**	**Input**	**Metric**	**Result (%)**	**Notes**
NTNU-HAR Children	Activity recognition (sitting, standing, walking, lying) from accelerometer data	Yes (70% training)	Macro F1-score	84.3	Adapter trained only; RL weights frozen
EEG-EMG Hand Exoskeleton	Motor intention classification (hand open/close) from EEG + EMG	Yes (70% training)	Accuracy	81.7	Same frozen-weights protocol
D4RL (halfcheetah-medium-v2)	Continuous control – halfcheetah locomotion	No (zero-shot)	Normalized score	68.5	Framework policy applied directly
D4RL (hopper-medium-replay-v2)	Continuous control – hopper hopping	No (zero-shot)	Normalized score	62.3	Same zero-shot setting

All reported metrics are averaged over 5-fold cross-validation with patient-level stratification. The 99% combined reward has a 95% confidence interval of [98.2%, 99.5%] (bootstrap, n=1000). Standard deviation across folds was ≤ 1.2% for all key metrics. An ablation study was conducted by removing three core components: without the meta-controller, convergence dropped to 85% and regret increased to 18%; without counterfactual reasoning, regret rose to 18% (from 10%); and without neuro-symbolic integration, global explainability fell to 91%. These results confirm that each component contributes significantly to the framework’s performance.

To assess generalization, we evaluated our framework on three public datasets without retraining the core RL components. For each dataset, we added a lightweight input adapter (a single linear layer) to project the new input features to the original model’s expected dimension. Only the adapter was trained on a 70% subset of each dataset; the RL model weights remained frozen. The remaining 30% was used for zero-shot testing. This setup preserves the framework’s learned decision policies while enabling evaluation on heterogeneous inputs.

Table 21 summarizes the tasks and results. For the two clinical datasets (NTNU-HARChildren and EEG-EMG Hand Exoskeleton), the adapter was trained on a 70% subset and tested on the remaining 30%. For D4RL (robotic control), we used the standard offline RL evaluation protocol without any adapter training, applying the framework’s policy directly to the benchmark’s state space. All public datasets are available for non-commercial research use under their respective licenses (CC0, Mendeley Data, and MuJoCo research license).

As shown in Table 22 below, our neuro-inspired hybrid RL framework benchmarks against contemporary AI systems, specialised healthcare RL applications, and human experts. Our approach achieves a superior combined reward of 99% and sample efficiency of 50% fewer samples while maintaining WHO/ICD-10 compliance, outperforming both generic deep RL methods, DQN: 85%, PPO: 89% and specialised clinical AI systems like Generative AI Diagnostics, 52.1% accuracy. Critically, our meta-control architecture demonstrates 95% edge-case convergence, resolving contextual limitations seen in Traditional CDSS 946 unaddressed factors 79 and systemic failures of the U.S. healthcare model highest amenable mortality^84^.

**Table 22:** Comparative Analysis Against State-of-the-Art Frameworks and Human Experts.

**Framework**	**Combined Reward**	**Sample Efficiency**	**Adaptability (Edge Cases)**	**Clinical Compliance**
**DQN **^15^	85%	Baseline	72%	Partial
**Proximal Policy Optimisation **^18^	89%	30% fewer	80%	Limited
**Generative AI Diagnostics **^85^	52.1% overall	15.8% ↓ vs. experts p=0.007	Limited black-box limitations	N/A
**Traditional CDSS **^86^	N/A	Variable rule-based constraints	946 context factors unaddressed	Chaotic decisions in 27/69 cases
**U.S. Health System **^84^	Last among 10 nations	2nd in care process, worst outcomes	Fragmented 26M uninsured	Highest amenable mortality
**Human-in-the-Loop ML **^87^	N/A	**88.63%** Parkinson’s detection	88% in sparse data	N/A
**WD3QNE, Sepsis **^88^	97.5% policy reward	83% → 97.5% survival uplift	Duelling + expert shaping	Sepsis reversal with high trust
**Sepsis **^89^	High Agent-based Env.	Not directly reported	Adaptive immunomodulation	Mortality was dramatically reduced in 500 patients
**ICU Sedation **^90^	Improved MAP control by 26%	Sedation accuracy ↑ 8% p<0.05	Personalised propofol/fentanyl dosing	Improved hemodynamic stability
**Ventilation **^91^	Mortality risk ↓, Oxygen ↑	Not directly reported	Real-time FiO₂/PEEP adjustments	Better oxygenation + reduced complications
**Diabetes, RL-DITR **^92^	High reward via glucose targets	MAE ≈ 1.10 U, glucose ↓ 11.1→8.6 mmol/L	Model-based insulin adjustment	Fewer hypo events, better glycemic control
**Parkinson’s **^93^	Not reported	Outperformed baseline dosing	Sensor-informed dosing	Reduced motor fluctuation
**Our Proposed Framework**	99%	50% fewer samples	95% convergence	WHO/ICD-10 aligned

While disease-specific RL implementations show promising results in sepsis 97.5% policy reward, ICU sedation 26% MAP improvement, and Parkinson’s motor fluctuation reduction, they lack the cross-domain adaptability and neurocognitive plausibility of our prefrontal-basal ganglia integration. Human experts remain the gold standard, 92% reward, but are constrained by scalability, a gap our framework bridges through bio-inspired sample efficiency.

## Conclusions

We presented a neuro-inspired hybrid RL architecture that dynamically combines MB planning with MF reflexes through a meta-controller (λ-arbitration), neuro-symbolic integration of WHO/ICD-10 guidelines, counterfactual reasoning, and ethical adaptability. Validated on a CP case study using multi-agent simulation (NetLogo) and offline classification (Weka), the framework achieved a 99% combined reward, 40% reduction in simulated falls, and 90% cross-domain generalisation accuracy. It outperformed DQN and PPO baselines in sample efficiency (50% fewer samples), robustness (≤10% error tolerance), and interpretability (99.8% local explainability). Ablation confirmed the contribution of each component: removing the meta-controller reduced convergence to 85%; removing counterfactual reasoning increased exploration regret to 18%; and removing neuro-symbolic integration lowered global explainability to 91%. External zero-shot validation on three public datasets (NTNU-HARChildren, EEG-EMG exoskeleton, D4RL) demonstrated generalisation to unseen tasks (macro F1 84.3%, accuracy 81.7%, D4RL scores 68.5/62.3). All metrics are reported with 95% confidence intervals and ≤1.2% standard deviation over 5-fold cross-validation. Future work includes clinical validation in a multi‑center CP trial, sub‑100 ms edge latency optimisation, counterfactual explainability for clinician‑in‑the‑loop training, adaptation to Parkinson’s and stroke, and federated learning for privacy‑preserving multi‑hospital training. These steps will transition the framework from simulation to real‑world deployment under WHO ethical guidelines.

## Data Availability

The dataset is not publicly available but can be accessed for research purposes by requesting data access from the University of Lahore Teaching Hospital (info@ucmd.uol.edu.pk). The hospital’s research committee approved the use of the dataset, which meets ethical standards. The dataset is fully anonymized.

## Abbreviations

PPO: Proximal policy optimisation
MF: Model-free
MB: Model-based
MAE: Mean absolute error
RMSE: Root mean square error
RAE: Relative absolute error
RRSE: Root relative squared error
CC: Correlation coefficient
MCC: Matthews correlation coefficient
ROC: Receiver operating characteristic
PRC: Precision-recall curve
BGRL: Basal GANGLIA-INSPIRED REINFORCEMENT LEARNING
ICH: Intracranial hemorrhage
BS: Body sensors
ICH: Intracranial hemorrhage
BS: Body sensors
UPWa: Uncomfortable places of the patient
LST: Long short-term memory
RL: Reinforcement learning
AI: Artificial intelligence
IoT: Internet of things
ML: Machine learning
DL: Deep learning
NLP: Natural language processing
CV: Computer vision
MDP: Markov decision process
DPWa: Dropped from a wheelchair
DBS: Deep brain stimulation
AIS: Acute ischemic stroke
ES: Environmental sensor
FPWa: Facial expression of patient
ES: Environmental sensor
FPWa: Facial expression of patient
EPWa: Empty wheelchair

## Materials availability

All materials used in this study are available from the corresponding author upon reasonable request.

## Code availability

The code implementing the proposed framework is available from the corresponding author upon reasonable request.
